# Increased NOX2 expression in astrocytes leads to eNOS uncoupling through dihydrofolate reductase in endothelial cells after subarachnoid hemorrhage

**DOI:** 10.3389/fnmol.2023.1121944

**Published:** 2023-03-30

**Authors:** Shu-Hao Miao, Sheng-Qing Gao, Hui-Xin Li, Yun-Song Zhuang, Xue Wang, Tao Li, Chao-Chao Gao, Yan-Ling Han, Jia-Yin Qiu, Meng-Liang Zhou

**Affiliations:** ^1^Department of Neurosurgery, Jinling Hospital, Jinling School of Clinical Medicine, Nanjing Medical University, Nanjing, China; ^2^Department of Neurosurgery, Jinling Hospital, Medical School of Nanjing University, Nanjing, China; ^3^Department of Gynecology, Women’s Hospital of Nanjing Medical University, Nanjing, China

**Keywords:** subarachnoid hemorrhage, acute vasoconstriction, endothelial nitric oxide synthase uncoupling, nicotinamide adenine dinucleotide phosphate oxidase 2 (NOX2), dihydrofolate reductase, neurovascular unit, acute cerebral ischemia

## Abstract

**Introduction:**

Endothelial nitric oxide synthase (eNOS) uncoupling plays a significant role in acute vasoconstriction during early brain injury (EBI) after subarachnoid hemorrhage (SAH). Astrocytes in the neurovascular unit extend their foot processes around endothelia. In our study, we tested the hypothesis that increased nicotinamide adenine dinucleotide phosphate oxidase 2 (NOX2) expression in astrocytes after SAH leads to eNOS uncoupling.

**Methods:**

We utilized laser speckle contrast imaging for monitoring cortical blood flow changes in mice, nitric oxide (NO) kits to measure the level of NO, and a co-culture system to study the effect of astrocytes on endothelial cells. Moreover, the protein levels were assessed by Western blot and immunofluorescence staining. We used CCK-8 to measure the viability of astrocytes and endothelial cells, and we used the H_2_O_2_ kit to measure the H_2_O_2_ released from astrocytes. We used GSK2795039 as an inhibitor of NOX2, whereas lentivirus and adeno-associated virus were used for dihydrofolate reductase (DHFR) knockdown *in vivo* and *in vitro*.

**Results:**

The expression of NOX2 and the release of H_2_O_2_ in astrocytes are increased, which was accompanied by a decrease in endothelial DHFR 12 h after SAH. Moreover, the eNOS monomer/dimer ratio increased, leading to a decrease in NO and acute cerebral ischemia. All of the above were significantly alleviated after the administration of GSK2795039. However, after knocking down DHFR both *in vivo* and *in vitro*, the protective effect of GSK2795039 was greatly reversed.

**Discussion:**

The increased level of NOX2 in astrocytes contributes to decreased DHFR in endothelial cells, thus aggravating eNOS uncoupling, which is an essential mechanism underlying acute vasoconstriction after SAH.

## 1. Introduction

Subarachnoid hemorrhage (SAH), primarily caused by ruptured aneurysm, is a common neurologically critical disease with high morbidity and mortality (Macdonald and Schweizer, 2017). As more than half of the patients died within 48 h of SAH and reversal of large-arterial spasm does not improve outcomes, early brain injury (EBI) that occurs within 72 h after SAH seems to be the main target of future research (Mayberg et al., 1994; Hop et al., 1997; Wang et al., 2012). One of the main features of EBI after SAH is a severe reduction in cerebral blood flow (CBF) (Adams et al., 1981; Schubert et al., 2009). Acute vasoconstriction, which has been observed in both experimental animals and patients after SAH, is a putative mechanism responsible for the decreased CBF in EBI (Bederson et al., 1995; Schubert et al., 2009; Yang et al., 2018). However, the mechanisms underlying the formation of acute cerebral vasoconstriction have not yet been fully elucidated, and effective treatments for SAH are still being explored (Ayer and Zhang, 2008).

Evidence suggests that oxidative stress is a factor leading to vasoconstriction after hemorrhage, but the specific mechanism by which oxidative stress regulates cerebral vasoconstriction still requires further study (Macdonald and Weir, 1994; Ayer and Zhang, 2008). As one of the main sources of reactive oxygen species (ROS) after SAH, nicotinamide adenine dinucleotide phosphate oxidase (NOX) has received extensive attention. To date, seven NOX isoforms (NOX1-5 and DUOX1-2) have been discovered (Cheng et al., 2001; Bedard and Krause, 2007). Among them, nicotinamide adenine dinucleotide phosphate oxidase 2 (NOX2) is highly expressed in cells throughout the central nervous system, including astrocytes (Cheng et al., 2001; Zhang et al., 2017). Several studies have found that increased NOX expression in the cerebrovascular system is associated with ROS-mediated vasospasm after SAH, while inhibition of NOX can reduce arterial constriction and improve CBF (Shin et al., 2002; Paravicini and Sobey, 2003; Miller et al., 2006). However, no previous study has determined the specific mechanism of acute vasoconstriction after SAH caused by increased NOX2 expression.

Reactive oxygen species, such as superoxide anion (O_2_^–^), and hydrogen peroxide (H_2_O_2_), are widely involved in cell signaling (Thannickal and Fanburg, 2000; Thannickal et al., 2000; Waghray et al., 2005). H_2_O_2_ is generally less reactive, longer-lived, and more stable than other ROS such as O_2_^–^ in most biological environments (Waghray et al., 2005). Normally, NOX2 produces O_2_^–^, which is converted to stable H_2_O_2_ by abundant, and ubiquitous extracellular and extracellular superoxide dismutase (Marklund, 1984; Jung et al., 2003). In addition, H_2_O_2_ is lipid soluble and can cross the plasma membrane to access targets in the cytoplasm to drive intracellular signaling cascades. Aquaporin (AQP) is a membrane protein that bidirectionally transports H_2_O and H_2_O_2_ across the membrane (Winterbourn, 2018). AQP is expressed in the plasma membrane and organelle membrane of cells throughout the animal kingdom (Lindskog et al., 2016; Nauseef, 2019). It is reasonable to guess that H_2_O_2_ released by astrocytes can enter endothelial cells.

It has been demonstrated that ROS generated by increased NOX2 expression contributes to brain endothelial dysfunction in angiotensin II-dependent hypertension, aging, and hypercholesterolemia (Iadecola et al., 2009; Miller et al., 2010; Chrissobolis et al., 2012). Nitric oxide (NO) is an important mediator in maintaining normal cerebrovascular function. Both clinical and experimental studies have shown a decline in NO after SAH (Schwartz et al., 2000; Sehba et al., 2000; Terpolilli et al., 2015, 2016). This early NO reduction is associated with endothelial dysfunction and is an important mechanism for early vasoconstriction in EBI (Sehba et al., 1999; Terpolilli et al., 2016). Endothelial nitric oxide synthase (eNOS) is a well-known member of the NOS enzyme family, which is highly expressed in endothelial cells, and eNOS is the main source of NO produced by endothelial cells (Förstermann and Sessa, 2012; Bendall et al., 2014). eNOS is a homodimer that can be converted from the dimer producing NO to the monomer producing superoxide anion in a process known as eNOS uncoupling (Förstermann and Sessa, 2012). Although the mechanism of eNOS uncoupling has not been fully elucidated, oxidation of the eNOS cofactor tetrahydrobiopterin (BH_4_) appears to be the main cause (Xia et al., 1998; Laursen et al., 2001). Dihydrofolate reductase (DHFR) catalyzes the regenerated BH_4_ from dihydrobiopterin (BH_2_), the oxidized form of BH_4_, in several cell types (Thöny et al., 2000). Moreover, studies have shown that DHFR deficiency leads to eNOS uncoupling by decreasing BH_4_ levels, thereby contributing to the development of vascular disease (Oak and Cai, 2007; Gao et al., 2009). Therefore, we hypothesized that this pathway may be involved in SAH leading to acute vasoconstriction.

Neurons, astrocytes, and endothelial cells are the main cells in the neurovascular unit, and they interact to ensure adequate levels of CBF in all areas of the brain (Abbott et al., 2006); this is the anatomical basis for the hypothesis that NOX2 in astrocytes may induce endothelial dysfunction through eNOS uncoupling after SAH, which was explored in this study.

## 2. Materials and methods

### 2.1. Animal preparation

All animal procedures were approved by the Jinling Hospital Animal Care and Use Committee and were conducted in accordance with the Guide for the Care and Use of Laboratory Animals published by the National Institutes of Health (Publication Nos. 80–23). Male C57/BL6 mice (6–8 w, 22–26 g) were purchased from Nanjing University (Nanjing, China). These mice were kept in an air-conditioned room with air humidity (50 ± 10%) and constant temperature (26 ± 2°C), under a 12 h light/dark cycle. All animals were provided with adequate food and water.

### 2.2. HA, hCMEC/D3, and *in vitro* SAH model

Human astrocytes (HA) cells were purchased from BeNa Culture Collection (Kunshan, China) and were cultured in a medium containing 90% high glucose DMEM, 10% fetal bovine serum (purchased from Gibco BRL, Grand Island, NY, USA). Human cerebral microvascular endothelial cells (hCMEC/D3) were purchased from ABM technology (Zhenjiang, China) and were cultured in a medium containing 89% Prigrow I Medium (TM001), 10% fetal bovine serum, and 1% penicillin-streptomycin. We created an *in vitro* SAH model by adding 20 μmol/L of oxyhemoglobin (OxyHb) (H2625, Sigma-Aldrich, St. Louis, MO, USA) to the above medium. The OxyHb we purchased from sigma is a qualified product derived from bovine blood. The hCMEC/D3 cell line is a stable, extensively characterized, and well-differentiated human brain endothelial cell line and it is suitable for research on blood–brain barrier function and the response of brain vascular endothelium to inflammation (Weksler et al., 2013).

### 2.3. *In vivo* SAH model

An experimental SAH model was established by injecting autologous arterial blood into the prechiasmatic cistern of the optic chiasm as described previously, with minor modifications (Deng et al., 2021). The mice were anesthetized using 100% O_2_ containing 2% isoflurane and fixed on a stereotaxic apparatus while maintaining anesthesia with 1% isoflurane. Under sterile conditions, we made an incision in the midline of the mouse scalp to expose the skull and subsequently drilled a 0.9 mm depth hole which located 4 mm in front of the bregma. A 27-gauge needle was passed through the burr hole at a 40° angle to the base of the skull. For the SAH group, 50 μl non-heparinized autologous femoral arterial blood was slowly injected into the prechiasmatic cisterna of mice using a syringe pump over 40 s. For the sham group, we injected an equal volume of normal saline. The 27-gauge needle was gently withdrawn 2 min after the blood/saline injections to prevent the backflow of blood and the leakage of cerebrospinal fluid. After sealing the hole using bone wax, the incision was sutured immediately. After injection, we monitored the mice for 40 min for recovery and then returned to their cages.

### 2.4. Experimental design

The schematic diagram of experimental design is demonstrated in Supplementary Figure 1. Our research is containing *in vivo* and *in vitro* parts. For the *in vivo* experiments, we first used laser speckle contrast imaging (LSCI) to compare the CBF of the sham and the 12 h groups after SAH (*n* = 6 each group). The mice in sham group were euthanized 12 h after operation (*n* = 6). We measured the time change of NOX2 expression in the temporal lobe of mice, and the change of NO and DHFR levels in endothelial cells extracted from the temporal lobe (12 h, 24 h, and 3 days; *n* = 6 each group). According to the results of the above experiments, we chose 12 h for further experiments. Then, 12 mice were randomly divided into sham group (*n* = 6), and 12 h group (*n* = 6), and collected their brain samples for immunofluorescence staining (Supplementary Figure 1A). To verify the hypothesized pathway, the mice were randomly divided into the following six groups: SAH, Vehicle + SAH, GSK + SAH(1), GSK + SAH(2), AAV-shDHFR + GSK + SAH, and AAV-shCtrl + GSK + SAH groups. For GSK/Vehicle + SAH group, two hours after the injection of GSK/Vehicle into the lateral ventricle of mice, we began to induce experimental SAH *in vivo*. For AAV-shCtrl/AAV-shDHFR + GSK + SAH group, AAV-shCtrl/AAV-shDHFR were administrated via tail vein injection 14 days before GSK injection. Before euthanasia of the mice in the above experiment, Neurological Score (*n* = 6 each group) and LSCI (*n* = 6 each group) were performed. As previously mentioned, their temporal lobe samples were gathered for NO content measurement (*n* = 6 each group), Western blot (*n* = 6 each group) and immunofluorescence staining (*n* = 6 each group) (Supplementary Figure 1B). For *in vitro* experiments, HA cells contained five groups: Control, OxyHb(1), OxyHb(2), Vehicle + OxyHb, and GSK + OxyHb groups. hCMEC/D3 cells contained the Control group and the OxyHb group. HA cells were co-cultured with hCMEC/D3 cells to explore the effect of astrocytes on endothelial cells, and the co-cultured cells were divided into six groups: AS + OxyHb, AS + Vehicle + OxyHb, AS + GSK + OxyHb(1), AS + GSK + OxyHb(2), LV-shCtrl + AS + GSK + OxyHb, and LV-shDHFR + AS + GSK + OxyHb groups. In the AS + Vehicle/GSK + OxyHb group, after using GSK/Vehicle alone on astrocytes for 2 h, we co-cultured astrocytes with endothelial cells and replaced the medium containing GSK/Vehicle with the medium containing OxyHb without GSK/Vehicle. We used DMSO as the vehicle in the *in vitro* experiments, and the dose of GSK2795039 was 20 μmol/ml. For LV-shCtrl/LV-shDHFR + AS + GSK + OxyHb groups, 48 h before co-culture, LV-shCtrl/LV-shDHFR were added to hCMEC/D3 cell culture medium. Vehicle/GSK were added to HA cell culture medium 2 h before co-culture. When HA cells were co-cultured with hCMEC/D3 cells, the culture medium containing OxyHb without GSK/Vehicle was replaced. The 6-well plates cultured cells were collected for Western blot (WB, *n* = 6 each group) while their corresponding culture medium of hCMEC/D3 cells was collected to evaluate the NO level (*n* = 6 each group). We also collected the medium of astrocytes to measure the amount of H2O2 released by astrocytes (*n* = 6 each group). In addition, we did immunofluorescence staining (IF)in 12-well plates cultured cells (*n* = 6 each group). We used 96-well plates to measure the viability of astrocytes and endothelial cells (*n* = 6 each group) (Supplementary Figure 1C).

### 2.5. Laser speckle contrast imaging

Laser speckle contrast imaging is based on blurring the interference pattern of scattered laser light through the flow of blood cells to instantaneously visualize blood perfusion in the circulation. We have been measured CBF and other experimental operations on mice on a constant temperature pad at 37°C, and we used air conditioning to maintain the room temperature at 28°C. Briefly, mice were anesthetized with gas anesthesia (2% isoflurane in 100% O_2_), their heads were fixed in a stereotaxic frame, and they were subjected to continuous inhalation of 1% isoflurane throughout the experiment. We made an incision in the midline of the mouse scalp. The blood flow was detected by a CCD camera, and image acquisition was performed using custom software. The same regions of interest were selected for different cortical samples and analyzed by two independent pathological researchers who were blinded to the groupings.

### 2.6. Isolation of the brain vasculature

We isolated cerebral blood vessels in the temporal lobe of brain, as previously published, with minor modifications (Bales et al., 2016). After cerebellum and olfactory bulb were removed, we homogenized the remaining brain tissue in 3 ml 4°C sucrose buffer (5 mM HEPES, 0.32 M sucrose, pH 7.4) with homogenizer. Then the homogenate of brain samples was centrifuged at 1,000 *g* for 12 min, after which the supernatant mainly containing neurons and the dense white myelin sheath on the top of the granules were discarded. Then we resuspended the particles in 3 ml 4°C sucrose solution and centrifuged them at 1,000 *g* for 12 min. The final pellet, which contained numerous cerebral blood vessels, was lysed in radioimmunoprecipitation assay which contained 1% phenylmethanesulfonyl fluoride (PMSF) for WB or NO content test.

### 2.7. Analysis of the intracellular NO production

Nitric oxide content of the extracted cortical blood vessels or culture medium was detected using a total nitric oxide assay kit (S0024, Beyotime). In brief, 80 μl of the extracted sample was added in a 96-well plate at room temperature, protected from light. Then, 7.5 μl of 2 mM nicotinamide adenine dinucleotide phosphate (NADPH), 15 μl of flavin adenine dinucleotide (FAD), and 7.5 μl of nitrate reductase were added to the sample sequentially, mixed, and incubated at 37°C for 30 min. Subsequently, 15 μl of lactate dehydrogenase (LDH) buffer and 15 μl of LDH were added, mixed, and incubated at 37°C for an additional 30 min. Finally, 50 μl of Griess reagent I and 50 μl of Griess reagent II were added, mixed, and incubated for 10 min at room temperature. The absorbance of each well was measured at 540 nm using a microplate reader. We quantified the protein content of the different samples and compared the NO levels at the same protein concentration.

### 2.8. Western blot

For Western blot, cells, and tissues were lysed in radioimmunoprecipitation assay buffer containing 1% PMSF and 1% phosphatase inhibitor cocktail (P5726, Sigma-Aldrich). The protein content was quantified using the Bicinchoninic acid protein assay (BCA) kit (P0011, Beyotime). The molecular weight of NOX2 is 60 kDa, the molecular weight of DHFR is 20 kDa, the molecular weight of eNOS monomer is 140 kDa, and the molecular weight of eNOS dimer is 280 kDa. The samples were separated by sodium dodecyl sulfate-polyacrylamide gel electrophoresis and transferred to polyvinylidene fluoride membranes. After the membranes were blocked in 5% skim milk at room temperature for 2 h, they were incubated with the following primary antibodies at 4°C overnight: anti-β-actin (1:5,000, 81115-1-RR, Proteintech, Chicago, IL, USA), anti-eNOS (1:500, 9572, Cell Signaling Technology, Danvers, MA, USA), anti-NOX2 (1:1,000, ab129068, Abcam) and anti-DHFR (1:2,000, ab124814, Abcam, Cambridge, MA, USA). The membranes were incubated using the appropriate secondary antibody (1:10,000, 7074, Cell Signaling Technology) for 1 h. The membrane is divided into distinct bands containing β-actin and other proteins of interest. Finally, the immunoreactive bands were detected using the enhanced chemiluminescence system. ImageJ software (National Institutes of Health, Bethesda, MD, USA) was used to analyze the relative densities of at least six independent experiments.

### 2.9. Immunofluorescence staining

The mouse brain sections (6 μm) were incubated with primary antibodies against NOX2 (1:200, ab133303, Abcam), DHFR (1:200, ab124814, Abcam), GFAP (1:200, 2389127, Invitrogen, Carlsbad, CA, USA), and CD31 (1:200, ab24590, Abcam) at 4°C overnight. GFAP is considered a standard marker of mature astrocytes and it is often used to label astrocytes in IF. CD31 is also commonly used to label endothelial cells in IF. So, we used CD31 as a marker to distinguish endothelial cells and GFAP as a marker to distinguish astrocytes. The brain slices were incubated using the corresponding goat anti-rabbit IgG antibodies (1:250, SA00009-2, Proteintech) or goat anti-mouse IgG (1:250, SA00013-1, Proteintech) for 1.5 h at room temperature. Subsequently, the brain sections were sealed with anti-fluorescence solution after incubated DAPI (C1005, Beyotime) for 6 min. Finally, the sections were observed and captured under the fluorescence microscope. Immunofluorescence staining of hCMEC/D3 and HA was similar to brain sections after fixing cells with 4% paraformaldehyde.

### 2.10. Co-cultures

The co-culture system consisted of lower and upper chambers separated by a selectively permeable membrane (Corning, Transwell 3450) with 0.4 μm-diameter pores. HA cells were plated in the upper chamber, hCMEC/D3 cells were plated in the lower chamber, and both were co-cultured to mimic neurovascular units.

### 2.11. Measuring cell viability

We measure cell viability using the Cell Counting Kit 8 (CCK-8). HA was seeded in 96-well plates at a density of 5,000 cells/well, and then treated as described in the experimental design for different groups. Because 96-well plates cannot be used for co-cultivation, we diluted different groups of hCMEC/D3 to the same multiple after treatment and seeded them in 96-well plates. After the cells were grown in the incubator for 24 h, they were washed 3 times with medium. Cells were subsequently incubated with CCK-8 solution and culture medium (diluted 1:10) at 37°C for 2 h, and then the absorbance was measured at 450 nm with a microplate reader.

### 2.12. Measurement of hydrogen peroxide

Hydrogen peroxide was measured *in vitro* using a hydrogen peroxide detection kit (S0038, Beyotime) and 96-well plates as previously described (Gao et al., 2022). Briefly, the media of astrocytes after different groups of treatments were collected. We added 50 μl of sample to each well, followed by 100 μl of detection reagent, mixed them and incubated them at room temperature for 30 min. The absorbance of each well was measured at 560 nm using a microplate reader, and then the hydrogen peroxide level in the medium was calculated according to the standard concentration curve.

### 2.13. Intracerebroventricular injection of GSK2795039

The mice were anesthetized using 100% O_2_ containing 2% isoflurane and their heads were fixed on a stereotaxic apparatus while maintaining anesthesia with 1% isoflurane. Under sterile conditions, an incision was made in the middle of the mouse scalp to expose the skull, and drills were used to drill 1.0 mm posterior to the bregma and 2.0 mm lateral to the bregma until the dura was penetrated. The vehicle-diluted GSK2795039 (HY-18950, MedChemExpress, NJ, USA) was injected vertically into the lateral ventricle through the hole at a flow rate of 2 μl/min using a syringe pump. For the vehicle group mice, an equal volume of vehicle was injected into the lateral ventricle. In the *in vivo* experiment, we mixed 10% DMSO, 40% PEG300, 5% Tween-80, and 45% saline as the vehicle, and the dose of GSK2795039 was 3 mg/kg. We kept the vehicle and GSK2795039 in a 37°C water bath before intraventricular injection. The needle was left in place after injection for 5 additional min before immediately sealing the drilled hole using bone wax. We observed the mice for 1 h during recovery before proceeding to the next experimental operation.

### 2.14. Lentiviral transfection

Lentiviral vectors which contained shRNA to knock down DHFR (the targeted sequence was CCUCUUCAGUAGAAGGUAATT; Lv-shDHFR) were obtained from Hanbio Biotechnology (Shanghai, China) and 30% proliferating fused hCMEC/D3 was incubated with the Lv-shDHFR mixture for 48 h before the next experiment.

### 2.15. Neurological scoring

Neurological function was assessed in mice using a modified Garcia Score by two investigators who were blinded to the grouping, using the following six separate tests: locomotion, symmetry of limb movements, forepaw extension, climbing, body proprioception, and response to touch (Gao et al., 2020). After calculating the total of the individual scores of all tests, the lowest neurobehavioral score was 3 points, which represented severely impaired neurological function, and the highest score was 18 points, which represented normal neurological function.

### 2.16. Statistical analysis

Statistical analysis was performed using Prism 8.02 (GraphPad Software, La Jolla, CA, USA). For each group, the values are expressed as the mean ± standard deviation (mean ± SD). Statistical differences were determined using one-way analysis of variance (ANOVA) when more than two groups were present. When ANOVA showed a significant difference, Tukey’s *post-hoc* test for multiple comparisons was applied to evaluate that difference. When only two groups were compared, student *t*-test was utilized. Statistical significance was inferred when the *p*-value was <0.05.

## 3. Results

### 3.1. CBF, NO content, NOX2, DHFR, and the ratio of eNOS monomer/dimer in the temporal lobes of mice after SAH

**FIGURE 1:**
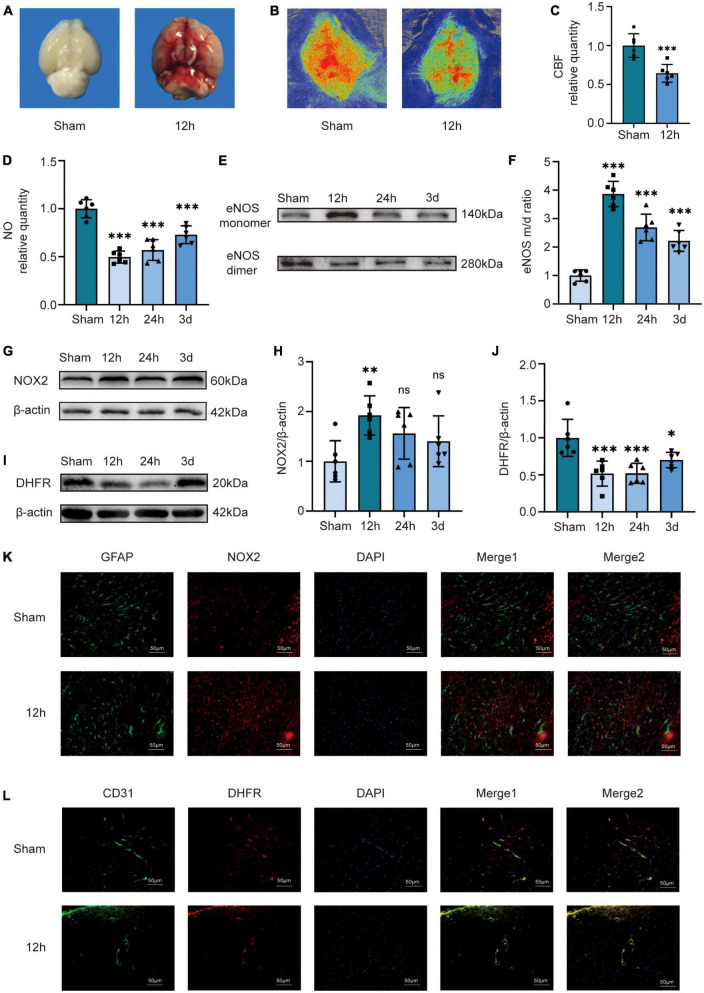
Cerebral blood flow, NO content, NOX2, DHFR, and the ratio of eNOS monomer/dimer in the temporal lobes of mice at different time courses after SAH in mice. **(A)** Compared to the Sham group, there was a larger amount of blood at the bottom of the brains of the 12 h group mice. **(B)** Representative LSCI images in these two groups. **(C)** Quantitative analysis of CBF in these two groups. Data are shown as mean ± SD (*n* = 6 mice each group, ^***^p < 0.001). **(D)** NO content in extracted endothelial cells of the Sham group and different time course groups after SAH. Data are shown as mean ± SD (*n* = 6 mice each group, ^***^p < 0.001). **(E,F)** Representative Western blot images and quantitative analysis of eNOS monomer and eNOS dimer expression in the Sham group and different time course groups after SAH by ImageJ. Data are shown as mean ± SD (*n* = 6 mice each group, ^***^p < 0.001). **(G,H)** Representative Western blot images and quantitative analysis of NOX2 expression in the temporal lobes of the Sham group and different time-course groups after SAH by ImageJ. Data are shown as mean ± standard deviation (*n* = 6 mice each group, ^**^p < 0.01, ns: no statistically significant difference). **(I,J)** Representative Western blot images and quantitative analysis for endothelial DHFR expression in the temporal lobe of mice in the Sham group and different time course groups after SAH by ImageJ. Data are shown as mean ± SD (*n* = 6 mice each group, *p < 0.05, ^***^p < 0.001, ns: no statistically significant difference). **(K)** Immunofluorescence images showed GFAP and NOX2 colocalization. All scale bars, 50 μm. **(L)** Immunofluorescence images showed CD31 and DHFR colocalization. All scale bars, 50 μm. CBF, cerebral blood flow; eNOS, endothelial nitric oxide synthase; NOX2, nicotinamide adenine dinucleotide phosphate oxidase 2; GFAP, glial fibrillary acidic protein; DHFR, dihydrofolate reductase.

We applied the prechiasmatic cistern injection model *in vivo*. We found that the bottom of the brain of mice in the 12 h group was filled with blood compared with the Sham group, demonstrating that the injection model well mimics the situation after SAH in humans (Figure 1A). We performed laser speckle examination on the mice in the Sham and 12 h groups (Figure 1B). The analysis results showed that compared to the sham group, the CBF of the mice in the 12 h group decreased significantly (Figure 1C). We examined the expression of DHFR, eNOS monomer, eNOS dimer, and NO content in cerebral vessels isolated from the temporal lobe. The results showed that compared to the Sham group, the endothelial NO content in the mice in the 12 h groups decreased significantly (Figure 1D). The results of Western blot showed that the eNOS monomer/dimer ratio was significantly increased at 12 h after SAH, which indicated that the uncoupling of eNOS was significantly increased 12 h after SAH (Figures 1E, F). The results of Western blot showed that NOX2 was significantly increased 12 h after SAH (Figures 1G, H). Therefore, we chose to conduct follow-up experiments 12 h after SAH. Likewise, we chose 12 h after SAH as the time point for Immunofluorescence staining analysis. Immunofluorescence staining showed the expression of NOX2 in astrocytes, which was notably increased 12 h after SAH (Figure 1K). The results of Western blot showed that compared to the sham group, DHFR decreased significantly at 12 h, 24 h, and 3 days after SAH, and the decrease was more obvious at 12 h (Figures 1I, J). Furthermore, Immunofluorescence staining showed that DHFR was mainly located in endothelial cells, and the expression of DHFR was significantly decreased 12 h after SAH (Figure 1L).

### 3.2. Release of H_2_O_2_ and NO, expression of NOX2 and DHFR, and the ratio of eNOS monomer/dimer in the SAH model *in vitro*

**FIGURE 2:**
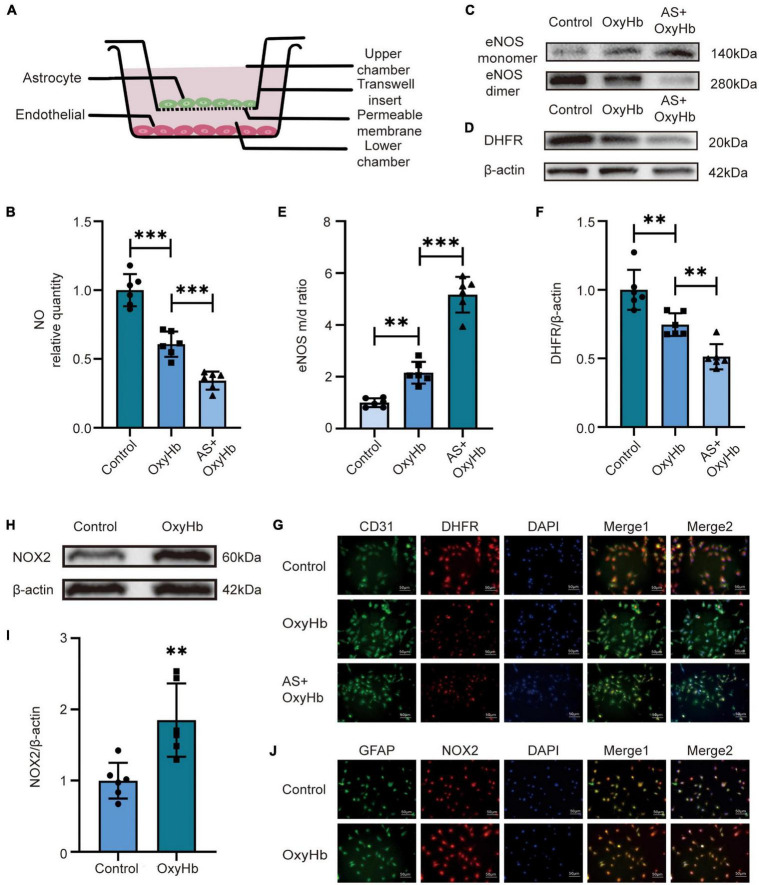
Alterations in NO release, NOX2 and DHFR expression, and eNOS monomer/dimer ratio in endothelial cells after SAH. **(A)** Diagram of co-culture of HA and hCMEC/D3 cells. **(B)** NO released by endothelial cells among different experimental groups. Data are shown as mean ± SD (*n* = 6 each group, ^***^p < 0.001, ns: no statistically significant difference). **(C,D)** Representative Western blot images and quantitative analysis of eNOS monomer and eNOS dimer expression by ImageJ. Data are shown as mean ± SD (*n* = 6 each group, ^**^p < 0.01, ^***^p < 0.001). **(E,F)** Representative Western blot images and quantitative analysis of endothelial DHFR expression by ImageJ. Data are shown as mean ± SD (*n* = 6 each group, ^**^p < 0.01). **(H,I)** Representative Western blot images and quantitative analysis of astrocytes NOX2 expression by ImageJ. Data are shown as mean ± SD (*n* = 6 each group, ^**^p < 0.01). **(G)** Immunofluorescence images showed the co-localization of CD31 and DHFR. All scale bars, 50μm. **(J)** Immunofluorescence images showed GFAP and NOX2 colocalization. All scale bars, 50 μm.

Subsequently, we used the SAH *in vitro* model to verify the above results. To explore the effect of astrocytes on endothelial cells, we co-cultured endothelial cells with astrocytes (Figure 2A). The data showed that the NO release from endothelial cells in the lower chamber medium in the OxyHb group was significantly lower than that in the control group and the NO in the medium of AS + OxyHb group was lower than that in the OxyHb group (Figure 2B). According to the results of Western blot, the OxyHb group showed a significant increase in the ratio of eNOS monomer/dimer in endothelial cells compared to the control group (Figures 2C, D). More importantly, the eNOS monomer/dimer ratio was significantly increased in endothelial cells in the AS + OxyHb group when compared to the OxyHb group (Figures 2C, D). The expression of DHFR also showed opposite trends to those of the eNOS monomer/dimer ratios (Figures 2E, F). Consistent with the results of Western blot, Immunofluorescence staining indicated that DHFR expression was significantly decreased in endothelial cells 12 h after SAH (Figure 2G). Taken together, these findings indicate that the presence of astrocytes increases endothelial cell eNOS uncoupling after SAH. Simultaneously, we also found that the expression of NOX2 in astrocytes increased significantly 12 h after adding OxyHb (Figures 2H, I). Moreover, in line with the results of Western blot, Immunofluorescence staining showed similar changes in NOX2 expression in astrocytes from diverse groups (Figure 2J).

### 3.3. Inhibition of NOX2 in astrocytes recouples eNOS by increasing DHFR expression in endothelial cells after OxyHb stimulation: Lv-shDHFR largely attenuated the protective effect of NOX2 inhibitors in the SAH model *in vitro*

**FIGURE 3:**
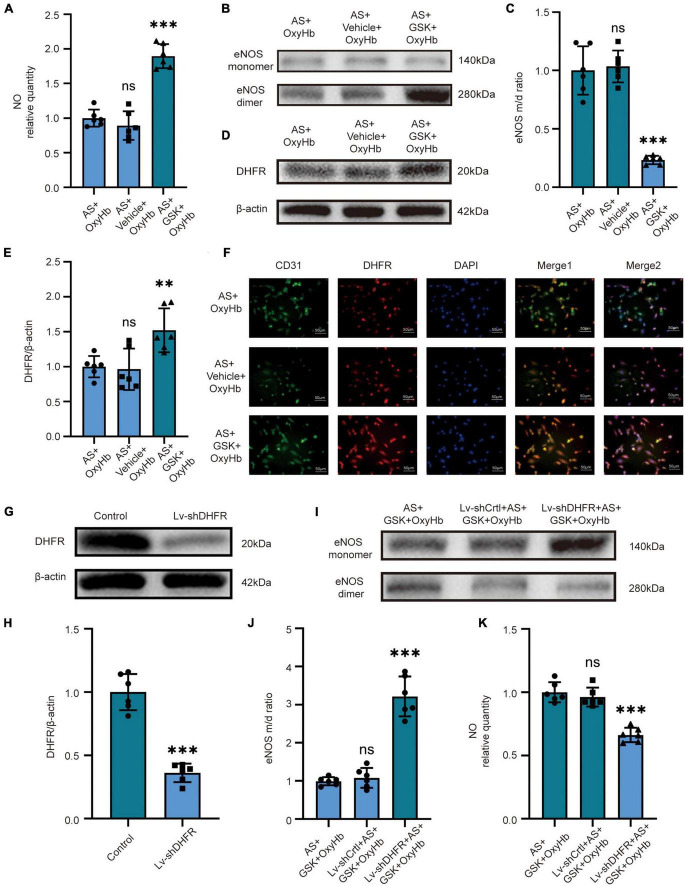
*In vitro*, inhibition of NOX2 in astrocytes after SAH recouples eNOS by increasing the expression of DHFR in endothelial cells. *In vitro*, LV-shDHFR largely attenuated the protective effect of NOX2 inhibitors after SAH. **(A)** NO released by cells among different experimental groups. Data are shown as mean ± SD (*n* = 6 each group, ^***^p < 0.001, ns: no significant difference). **(B,C)** Representative Western blot images and quantitative analysis of eNOS monomer and eNOS dimer expression by ImageJ. Data are shown as mean ± SD (*n* = 6 each group, ^***^p < 0.001, ns: no statistically significant difference). **(D,E)** Representative Western blot images and quantitative analysis of endothelial DHFR expression by ImageJ. Data are shown as mean ± SD (*n* = 6 each group, ^**^p < 0.01, ns: no statistically significant difference). **(F)** Immunofluorescence images showed CD31 and DHFR colocalization. All scale bars, 50μm. **(G,H)** Representative Western blot images and quantitative analysis of endothelial DHFR expression by ImageJ. Data are shown as mean ± SD (*n* = 6 each group, ^***^p < 0.001). **(I,J)** Representative Western blot images and quantitative analysis of eNOS monomer and eNOS dimer expression by ImageJ. Data are shown as mean ± SD (*n* = 6 each group, ^***^p < 0.001). **(K)** NO released by cells among different experimental groups. Data are shown as mean ± SD (*n* = 6 each group, ^***^p < 0.001, ns: no statistically significant difference).

Because the above *in vivo* and *in vitro* results were similar, we set out to validate our pathway using NOX2 inhibitors and Lv-shDHFR. To further verify the effect of NOX2 in astrocytes on endothelial cells, we used GSK2795039 to inhibit NOX2 in astrocytes and co-cultured astrocytes with endothelial cells. After adding OxyHb for 12 h, we found no significant difference in NO content, eNOS monomer/dimer ratio, and DHFR expression in the AS + OxyHb and AS + vehicle + OxyHb groups but surprisingly found that compared to the AS + OxyHb group, the NO content was significantly increased in the AS + GSK + OxyHb group (Figure 3A). The results of Western blot showed that compared to the AS + OxyHb group, the eNOS monomer/dimer ratio in the AS + GSK + OxyHb group was significantly decreased (Figures 3B, C) and the expression of DHFR was significantly increased (Figures 3D, E). Moreover, compared to the AS + OxyHb group, the DHFR expression was significantly increased in endothelial cells in the AS + GSK + OxyHb group (Figure 3F). We found that there was no significant difference in the viability of astrocytes in the OxyHb group, Vehicle + OxyHb group, and GSK + OxyHb group (Supplementary Figure 2A). However, we found that the viability of endothelial cells in the AS + GSK + OxyHb group was significantly increased compared with the AS + OxyHb group, while there was no significant difference in the viability of endothelial cells between the AS + OxyHb group and the AS + Vehicle + OxyHb group (Supplementary Figure 2B). We found that compared with the Control group, the H_2_O_2_ released from astrocytes in the medium in the OxyHb group was significantly increased, while compared with the OxyHb group, the H_2_O_2_ in the GSK + OxyHb group was significantly decreased (Supplementary Figure 3). This proves that a large part of H_2_O_2_ released by astrocytes after SAH is mediated by NOX2. These findings demonstrate that eNOS and DHFR are downstream effectors of NOX2 in astrocytes.

We demonstrated the knockdown efficacy of Lv-shDHFR in endothelial cells by Western blot (Figures 3G, H). The results showed no significant difference in NO content or eNOS monomer/dimer ratio in the AS + GSK + OxyHb and Lv-shCtrl + AS + GSK + OxyHb groups. The Western blot results showed that compared to the AS + GSK + OxyHb group, the eNOS monomer/dimer ratio was significantly increased in the Lv-shDHFR + AS + GSK + OxyHb group (Figures 3I, J). Compared to the AS + GSK + OxyHb group, the amount of NO released by endothelial cells was significantly decreased in the Lv-shDHFR + AS + GSK + OxyHb group (Figure 3K). These results demonstrate that knockdown of DHFR reduces the protective effect of GSK2795039. These results also confirmed the existence of our conjectured pathway.

### 3.4. NOX2 inhibitor increased CBF in mice by recoupling eNOS after SAH

**FIGURE 4:**
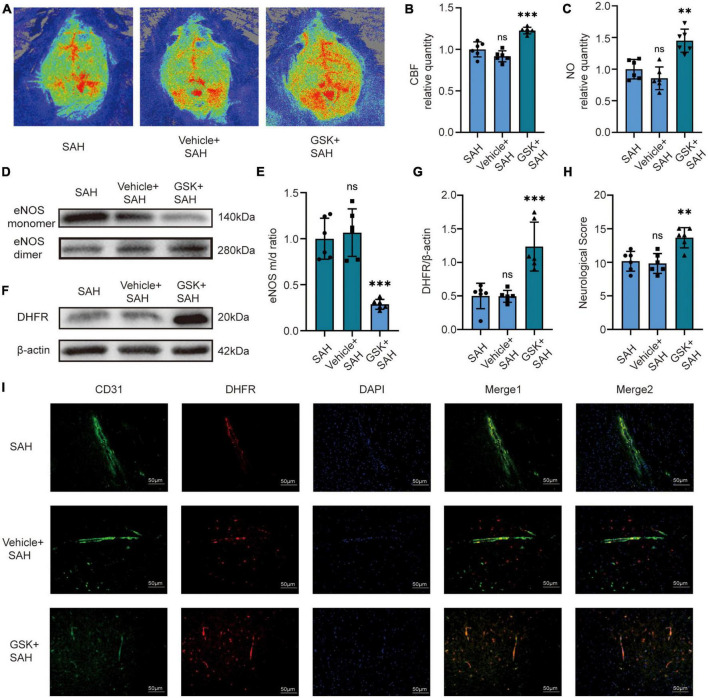
*In vivo*, NOX2 inhibitors increased CBF in mice by recoupling eNOS after SAH. **(A)** Representative LSCI images among different experimental groups. **(B)** Quantitative analysis of CBF among different experimental groups. Data are shown as mean ± SD (*n* = 6 mice each group, ^***^p < 0.001, ns, no statistically significant difference). **(C)** NO content in extracted endothelial cells among different experimental groups. Data are shown as mean ± SD (*n* = 6 mice each group, ^**^p < 0.01, ns: no statistically significant difference). **(D,E)** Representative Western blot images and quantitative analysis of eNOS monomer and eNOS dimer expression by ImageJ. Data are shown as mean ± SD (*n* = 6 mice each group, ^***^p < 0.001). **(F,G)** Representative Western blot images and quantitative analysis of endothelial DHFR expression by ImageJ. Data are shown as mean ± SD (*n* = 6 mice each group, ^***^p < 0.001). **(H)** Modified Garcia Score among different experimental groups. Data are shown as mean ± SD (*n* = 6 mice each group, ^**^p < 0.01, ns: no statistically significant difference). **(I)** Immunofluorescence images showed CD31 and DHFR colocalization. All scale bars, 50μm.

We then set out to validate our pathway *in vivo*. For *in vivo* experiments, we injected GSK2795039 into the ventricles of mice to inhibit NOX2. The laser speckle diagram showed that there was no significant difference in CBF between the SAH group and the vehicle + SAH group, however, compared to the SAH group, mice in the GSK + SAH group had increased CBF (Figures 4A, B). Simultaneously, we found no significant difference in endothelial NO content, eNOS monomer/dimer ratio, and DHFR expression in the SAH and vehicle + SAH groups. However, compared to the SAH group, the endothelial NO content was significantly increased in the GSK + SAH group (Figure 4C). Moreover, as shown in the cell experiments, compared to the vehicle + SAH group, the ratio of eNOS monomer/dimer and the expression of DHFR were increased significantly in the GSK + SAH group (Figures 4D–G). After injection of GSK2795039, the deteriorating neurological function was also significantly relieved (Figure 4H). Moreover, the results of Immunofluorescence staining showed that compared to the vehicle group, the NOX2 inhibitor significantly increased the expression of DHFR in endothelial cells (Figure 4I). Similar to the results of cell experiments, these findings also demonstrate that eNOS and DHFR are downstream effectors of NOX2.

### 3.5. Knockdown of DHFR in mice largely attenuates the protective effect of NOX2 inhibitors after SAH

**FIGURE 5:**
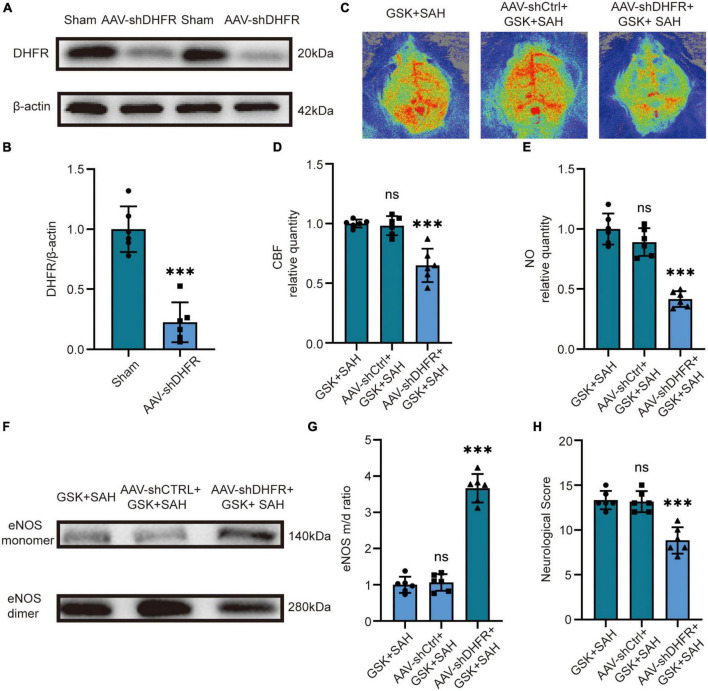
*In vivo*, knockdown of DHFR partially counteracted the effect of NOX2 inhibitors on relieving acute cerebral ischemia in SAH. **(A,B)** Representative Western blot images and quantitative analysis of endothelial DHFR expression by ImageJ. Data are shown as mean ± SD (*n* = 6 mice each group, ^***^p < 0.001). **(C)** Representative LSCI images among different experimental groups. **(D)** Quantitative analysis of CBF among different experimental groups. Data are shown as mean ± SD (*n* = 6 mice each group, ^***^p < 0.001, ns: no statistically significant difference). **(E)** NO content in extracted endothelial cells among different experimental groups. Data are shown as mean ± SD (*n* = 6 mice each group, ^***^p < 0.001, ns: no statistically significant difference). **(F,G)** Representative Western blot images and quantitative analysis of eNOS monomer and eNOS dimer expression by ImageJ. Data are shown as mean ± SD (*n* = 6 mice each group, ^***^p < 0.001). **(H)** Modified Garcia Score among different experimental groups. Data are shown as mean ± SD (*n* = 6 mice each group, ^***^p < 0.001, ns: no statistically significant difference).

We first knocked down DHFR by injecting an adeno-associated virus (AAV) into the tail vein of mice, and the knockdown efficacy of AAV-shDHFR was demonstrated by Western blot (Figures 5A, B). The laser speckle diagram demonstrates no significant difference in CBF between the GSK + SAH and AAV-shCtrl + GSK + SAH groups, however, compared to the GSK + SAH group, mice in the AAV-shDHFR + GSK + SAH group had increased CBF (Figures 5C, D). We also found no significant difference in endothelial NO content and eNOS monomer/dimer ratio between the GSK + SAH and AAV-shCtrl + GSK + SAH groups. The data showed that compared to the GSK + SAH group, the endothelial NO content was significantly decreased in the GSK + AAV-shDHFR + SAH group (Figure 5E). The Western blot results showed that the eNOS monomer/dimer ratio in the GSK + AAV-shDHFR + SAH group was significantly increased compared to that in the GSK + SAH group (Figures 5F, G). The protective effect of GSK2795039 in attenuating neurological deficits was also abolished by DHFR knockdown in endothelial cells (Figure 5H). This result proves that DHFR is a downstream factor of NOX2, and its knockdown counteracts the partial alleviation of acute cerebral ischemia in SAH by NOX2 inhibitors.

## 4. Discussion

**FIGURE 6:**
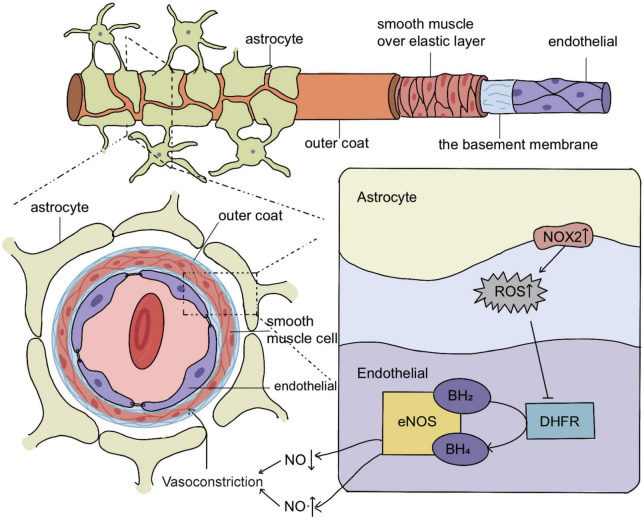
Schematic representation of the molecular mechanism of eNOS uncoupling in acute vasoconstriction after subarachnoid hemorrhage (SAH). In SAH, the increased expression of NOX2 in astrocytes causes the cells to release excess H_2_O_2_, and H_2_O_2_ enters the endothelial cells to reduce the expression of DHFR, which leads to the decrease of BH_4_, a key cofactor of eNOS. The decrease of BH_4_ increases the uncoupling of eNOS and reduces its production of NO. This leads to acute cerebral ischemia after SAH, most likely by mediating acute vasoconstriction.

In the present study, we found that NOX2 of astrocytes was increased after experimental SAH, contributing to the decreased expression of endothelial DHFR and aggravated eNOS uncoupling, which was an essential mechanism underlying the acute cerebral ischemia after SAH. The mechanism diagram is shown in Figure 6.

We applied the prechiasmatic cistern injection model *in vivo* by infusing quantitative amounts of fresh autologous blood into the subarachnoid space under appropriate pressure. This closely mimics the situation in humans with SAH where blood fills the subarachnoid space and surrounds the main conducting arteries (Sabri et al., 2009). The intravascular perforation model has the disadvantages of difficulty to control bleeding volume and high mortality (up to 50%) (Bederson et al., 1995). While the injection model has a lower mortality rate, the mortality rate of each group of mice in our *in vivo* experiments is listed in Supplementary Table 1. Moreover, using the injection model, we can ensure a constant infusion pressure as well as a quantitative blood volume (Matz et al., 2000; Hansen-Schwartz et al., 2003). It ensures that the bleeding intensity and the damage caused by each experiment are repeatable, which is very important for the accuracy of our measurement of CBF and other experimental results (Sehba et al., 2012).

Therapeutic strategies that inhibit ROS-generating enzymes or scavenge ROS alleviate vasoconstriction in animal models of SAH (Horky et al., 1998; McGirt et al., 2002b). Among various ROS sources, such as NOX, cyclooxygenase, and xanthine synthase, NOX is considered as the main source of ROS in the brain (Ayer and Zhang, 2008). In experimental models of cerebral ischemia, NOX2 knockout mice have smaller cerebral infarct size and less blood-brain barrier disruption compared to wild-type mice (DiNapoli et al., 2008; Cairns et al., 2012). Furthermore, hyperbaric oxygen induces neuroprotection after SAH by downregulating NOX2 and subsequently reducing oxidative stress (Ostrowski et al., 2006b,c). We demonstrated that NOX2 in astrocytes was elevated 12 h after SAH, which was similarly demonstrated by Zhang et al. (2017) who demonstrated that silencing NOX2 inhibited neuronal apoptosis after SAH. We showed that elevated NOX2 expression in astrocytes contributed to acute cerebral ischemia after SAH. A previous study demonstrated that the application of the NOX inhibitor diphenyleneiodonium in the cisterna magna can partially attenuate cerebral vasospasm (Shin et al., 2002). Our experiments also demonstrated that the application of the NOX2 inhibitor, GSK2795039, effectively alleviates acute cerebral ischemia.

In the brain, astrocytes produce and release various molecular mediators, such as prostaglandins and arachidonic acid, that regulate vessel diameter (Iadecola and Nedergaard, 2007; Gordon et al., 2011; Liu and Chopp, 2016). It has been shown in the literature that NADPH oxidase autocrine and paracrine H_2_O_2_ play a signaling role in various biological environments (Lambeth and Neish, 2014; Breitenbach et al., 2018). Similarly, it has been documented that the ROS produced by NADPH oxidase in the adventitia enters the inner side of the blood vessel and causes intimal hypertrophy, and other experiments have shown that the diffusion of H_2_O_2_ produced by the adventitia causes contraction of the abdominal aorta in mice (Wang et al., 1998; Di Wang et al., 1999; Rey et al., 2002). We also demonstrated that astrocytes can release H_2_O_2_ into the medium, which may enter endothelial cells through diffusion and AQP. Our results and these findings above suggest that astrocytes can release H_2_O_2_ into endothelial cells to mediate eNOS uncoupling.

There is evidence that persistent oxidative stress leads to the uncoupling of eNOS, reducing its production of NO, while increasing evidence suggests that NOX plays a key role in the phenomenon of eNOS uncoupling (Landmesser et al., 2003; Förstermann and Münzel, 2006; Förstermann, 2008, 2010). It has been demonstrated that NOX activation occurs upstream of eNOS uncoupling in endothelial cells in diabetic mice and ischemia/reperfusion-injured hearts (Chalupsky and Cai, 2005; Oak and Cai, 2007). We also demonstrated that inhibition of NOX2 after SAH resulted in reduced uncoupling of eNOS.

Numerous experiments have shown that endothelial DHFR deficiency leads to decreased BH_4_ and eNOS uncoupling in many cardiovascular diseases, including hypertension, aortic aneurysm, and diabetic vascular complications (Gao et al., 2012; Youn et al., 2012; Li et al., 2019). Another previous experiment showed that restoring DHFR can recouple eNOS to lower blood pressure (Li et al., 2015). We demonstrate that, after SAH, there is a decrease in DHFR expression in endothelial cells that leads to eNOS uncoupling, which subsequently leads to acute cerebral ischemia.

Both our research and that of several other studies have demonstrated an increase in eNOS uncoupling after SAH by a decrease in NO and an increase in the eNOS monomer/dimer ratio (Sabri et al., 2011a,b). It has been shown that 3 h after SAH, eNOS-deficient mice have less microvascular perfusion and increased microvascular spasm compared to wild-type mice (Lenz et al., 2021). However, SAH treatment with simvastatin, a compound that increases eNOS expression in cerebral blood vessels, alleviates cerebral vasospasm in a mouse model of SAH (McGirt et al., 2002a). An article from our laboratory and our study showed that DHFR was significantly decreased at 12 h, 24 h, and 3 days after SAH while eNOS uncoupling was significantly increased at 12 h, 24 h, and 3 days after SAH. This may indicate that eNOS uncoupling occurs before 12 h after SAH, which may be a direction worthy of further investigation (Gao et al., 2022). Our study demonstrated that inhibition of NOX2 decreased eNOS uncoupling after SAH, resulting in elevated NO and reduced acute cerebral ischemia in mice. Taken together, these findings provide evidence that eNOS coupling plays an important role in maintaining proper vascular function after SAH.

Cell interactions in neurovascular units are complex, with one study showing that SAH increases the amplitude of astrocyte terminal Ca^2+^ oscillations and induces parenchymal arteriole constriction (Pappas et al., 2015). We used co-culture techniques to explore the role of astrocytes in endothelial cells and to link oxidative stress and endothelial dysfunction after SAH. Our study is the first to demonstrate that in SAH, elevated NOX2 expression in astrocytes leads to decreased DHFR expression and subsequent eNOS uncoupling in endothelial cells.

At present, there are two methods for measuring cerebral perfusion: (1) The more intuitive methods for evaluating cerebral perfusion include traditional measurement of Intracranial pressure (ICP)/cerebral perfusion pressure (CPP), optical methods including LSCI, transcranial Doppler (TCD), CT perfusion imaging (PCT), For example, PCT mainly conducts qualitative and quantitative analysis through hemodynamic parameters such as cerebral blood volume and mean transit time. (2) Indirect assessment of CBF adequacy by monitoring global or regional oxygenation and metabolism (jugular venous oxygen saturation, regional cerebral oxygen saturation, and microdialysis) (Artru et al., 2004; Rao and Durga, 2011; Mir et al., 2014; Deffieux et al., 2018). LSCI provides high-resolution images with a large field of view, so we chose LSCI to directly measure CBF in mice (Dunn, 2012). ICP rises with the release of blood in the brain after SAH, rising to the level of mean arterial pressure within 1 min after SAH. The ICP then drops above but close to its physiological level within minutes (Grote and Hassler, 1988). Concurrently, CPP decreases, and CBF decreases, which leads to severe cerebral ischemia (Cahill et al., 2006; Ostrowski et al., 2006a). CPP is the difference between mean arterial pressure and ICP. Both ICP and CPP are important indicators related to CBF. CBF is determined by CPP and cerebrovascular resistance (CVR). CVR is determined by the diameter and length of blood vessels and the viscosity of blood (Fan et al., 2022). It has been widely documented that the presence of acute vasoconstriction in SAH can reduce CBF by reducing the diameter of blood vessels.

The temporal order in which early microvascular constriction occurs has been roughly elucidated in experimental studies using histological techniques and *in vivo* imaging (Plesnila, 2013). As early as 5 min after SAH, the small blood vessels at the base of the brain begin to constrict. Subsequently, intraparenchymal and pial microvessels contract sharply within 24 h of SAH (Bederson et al., 1998; Sun et al., 2009; Friedrich et al., 2012). In studies observing the cerebral vessels in patients and experimental animals within hours after SAH, they showed that arteriolar constriction reduced CBF by 60–80% (Uhl et al., 2003; Pennings et al., 2004). Our experiments also demonstrated a sharp decrease in CBF at 12 h after SAH by measuring CBF in mice using LSCI.

In EBI after SAH, not only early vasoconstriction but also microvascular thrombus existed. Both mechanisms lead to an early decline in CBF, but studies have shown a significant correlation between vasoconstriction and microvascular thrombosis (Clarke et al., 2020). Microvascular thrombosis almost only occurs in small arteries with significant constriction. Dynamic imaging of arteriolar constriction then demonstrates vessel constriction within seconds after SAH, leading to secondary thrombus formation (Friedrich et al., 2012). Another study showed that no microvascular thrombosis was found in unconstricted blood vessels (Sabri et al., 2012). These results may indicate that vasoconstriction leads to thrombus formation, rather than thrombus occlusion of microvessels causing their secondary constriction. Further, studies have pointed out that 70% of arterioles in the brain after SAH constricted and the proportion of blood vessel diameter decreased by more than 30% (Friedrich et al., 2012). A 30% decrease in the diameter of blood vessels leads to an approximately 80% decrease in CBF, according to the Hagen–Poiseuille law. This is enough to cause cerebral ischemia. At the same time, because half of the observed small arteries have a stronger degree of constriction, and the constriction of small arteries in blood vessels has the greatest impact on blood flow, the number and degree of constriction of small arteries can indicate that a large part of the decline in CBF is due to vasoconstriction (Friedrich et al., 2012). The current study shows that reduction of endothelial DHFR mainly results in reduction of NO, a well-known factor promoting vasodilation, in several diseases including SAH (Gao et al., 2009, 2022). So, we can speculate that a large part of the decrease in blood flow caused by the decrease in NO in our experiment is mediated by vasoconstriction.

However, our study has some limitations. The types of astrocytes we used were mixed. We did not repeat the validation using fibrous or protoplasmic astrocytes or different cell lines. It has been reported that the NOX2 protein level of astrocytes in the perihematomal brain tissue of SAH patients is increased, and it has been proved that the increase of NOX2 will produce excessive ROS in many types of cells, and our experiments and supplementary experiments also demonstrated the increased expression of NOX2 and the increased release of ROS in astrocytes (Marchetto et al., 2008; Zhang et al., 2017; Nauseef, 2019). At the same time, both fibrous and protoplasmic astrocytes have been shown to wrap around cerebral blood vessels, so they both may secrete ROS into endothelial cells (Bozic et al., 2021). Many articles have proved that the increase of ROS in endothelial cells will lead to the decrease of DHFR and the uncoupling of eNOS (Li et al., 2019; Gao et al., 2022). So even though the degree of DHFR reduction may vary with increasing ROS content released by different cell lines or astrocyte types, this would not rebut our conclusions. We will use different types of astrocytes to verify the conclusion in future work.

Both NOX2 from astrocytes and uncoupled eNOS from endothelial cells can release H_2_O_2_, and limited by technical conditions, we could not distinguish the specific source of H_2_O_2_ in the medium of co-cultivation after SAH. Meanwhile, the measurement of ROS *in vivo* remains a challenge, which may require confocal imaging techniques and ESR analysis. However, the low levels and relatively short lifetime of ROS make measurement difficult, and several processes and side effects associated with laser exposure (such as photobleaching or opposite effects of photoactivation) can make data difficult to analyze and interpret (Fuloria et al., 2021). These existing technical problems still need to be solved, and we will study how to solve these problems in future research.

## 5. Conclusion

In conclusion, our study revealed a novel mechanism by which oxidative stress leads to acute cerebral ischemia after SAH, that is, elevated NOX2 expression in astrocytes leads to eNOS uncoupling through endothelial DHFR downregulation. This signaling pathway leads to a decrease in NO, which contributes to acute cerebral ischemia after SAH most likely by causing acute vasoconstriction. This signaling cascade may represent a pervasive mechanism of eNOS uncoupling under pathophysiological conditions associated with oxidative stress. These findings are also highly translational in facilitating the development of drugs for treating SAH.

## Data availability statement

The raw data supporting the conclusions of this article will be made available by the authors, without undue reservation.

## Ethics statement

The animal study was reviewed and approved by the Jinling Hospital Animal Care and Use Committee.

## Author contributions

M-LZ, TL, Y-LH, and S-HM contributed to conception and design of the study. S-HM, S-QG, H-XL, Y-SZ, XW, and J-YQ conducted the experiments. M-LZ, C-CG, and TL performed the statistical analysis. S-HM wrote the first draft of the manuscript. S-QG and H-XL wrote the sections of the manuscript. All authors contributed to manuscript revision, read, and approved the submitted version.

## Supplementary material

Supplementary Figure 1 Experimental schematic *in vivo* and *in vitro*. **(A)** Experimental design diagram of the Sham group and the 12 h, 24 h, and 3 days groups after SAH. **(B)** Experimental design diagram to explore signaling pathways *in vivo.* **(C)** Experimental design diagram to explore signaling pathways *in vitro*. SAH, subarachnoid hemorrhage; OxyHb, oxyhemoglobin; AS, astrocyte; WB, Western blot; IF, immunofluorescence staining; H_2_O_2_, hydrogen peroxide; NO, nitric oxide; LSCI, laser speckle contrast imaging; GSK, GSK2795039; LV, lentivirus; AAV, adeno-associated virus.

Supplementary Figure 2 The viability of different groups of astrocytes and endothelial cells. **(A)** Astrocyte viability among different experimental groups. Data are shown as mean ± SD (*n* = 6 each group, ns: no statistically significant difference). **(B)** Endothelial cell viability among different experimental groups. Data are shown as mean ± SD (*n* = 6 each group, ^***^*p* < 0.001, ns: no statistically significant difference).

Supplementary Figure 3 The content of H_2_O_2_ released by astrocytes in the medium. **(A)** H_2_O_2_ released by astrocytes among different experimental groups. Data are shown as mean ± SD (*n* = 6 each group, ^***^*p* < 0.001). **(B)** H_2_O_2_ released by astrocytes among different experimental groups. Data are shown as mean ± SD (*n* = 6 each group, ^***^*p* < 0.001, ns: no statistically significant difference).

## References

[B1] Abbott N. J. Rönnbäck L. Hansson E. (2006). Astrocyte-endothelial interactions at the blood-brain barrier. *Nat. Rev. Neurosci.* 7 41–53. 10.1038/nrn1824 16371949

[B2] Adams H. P. Jr. Kassell N. F. Torner J. C. Nibbelink D. W. Sahs A. L. (1981). Early management of aneurysmal subarachnoid hemorrhage. A report of the Cooperative Aneurysm Study. *J. Neurosurg.* 54 141–145. 10.3171/jns.1981.54.2.0141 7005404

[B3] Artru F. Dailler F. Burel E. Bodonian C. Grousson S. Convert J. (2004). Assessment of jugular blood oxygen and lactate indices for detection of cerebral ischemia and prognosis. *J. Neurosurg. Anesthesiol.* 16 226–231. 10.1097/00008506-200407000-00007 15211160

[B4] Ayer R. E. Zhang J. H. (2008). Oxidative stress in subarachnoid haemorrhage: Significance in acute brain injury and vasospasm. *Acta Neurochir. Suppl.* 104 33–41. 10.1007/978-3-211-75718-5_7 18456995 PMC2743548

[B5] Bales K. R. O’neill S. M. Pozdnyakov N. Pan F. Caouette D. Pi Y. (2016). Passive immunotherapy targeting amyloid-β reduces cerebral amyloid angiopathy and improves vascular reactivity. *Brain* 139 563–577. 10.1093/brain/awv313 26493635

[B6] Bedard K. Krause K. H. (2007). The Nox family of Ros-generating Nadph oxidases: Physiology and pathophysiology. *Physiol. Rev.* 87 245–313. 10.1152/physrev.00044.2005 17237347

[B7] Bederson J. B. Germano I. M. Guarino L. (1995). Cortical blood flow and cerebral perfusion pressure in a new noncraniotomy model of subarachnoid hemorrhage in the rat. *Stroke* 26 1086–1091; discussion 1091–1092. 10.1161/01.STR.26.6.1086 7762027

[B8] Bederson J. B. Levy A. L. Ding W. H. Kahn R. Diperna C. A. Jenkins A. L. III (1998). Acute vasoconstriction after subarachnoid hemorrhage. *Neurosurgery* 42 352–360; discussion 360–362. 10.1097/00006123-199802000-00091 9482187

[B9] Bendall J. K. Douglas G. Mcneill E. Channon K. M. Crabtree M. J. (2014). Tetrahydrobiopterin in cardiovascular health and disease. *Antioxid. Redox Signal.* 20 3040–3077. 10.1089/ars.2013.5566 24294830 PMC4038990

[B10] Bozic I. Savic D. Lavrnja I. (2021). Astrocyte phenotypes: Emphasis on potential markers in neuroinflammation. *Histol. Histopathol.* 36 267–290. 33226087 10.14670/HH-18-284

[B11] Breitenbach M. Rinnerthaler M. Weber M. Breitenbach-Koller H. Karl T. Cullen P. (2018). The defense and signaling role of NADPH oxidases in eukaryotic cells : Review. *Wien. Med. Wochenschr.* 168 286–299. 10.1007/s10354-018-0640-4 30084091 PMC6132560

[B12] Cahill J. Calvert J. W. Zhang J. H. (2006). Mechanisms of early brain injury after subarachnoid hemorrhage. *J. Cereb. Blood Flow Metab.* 26 1341–1353. 10.1038/sj.jcbfm.9600283 16482081

[B13] Cairns B. Kim J. Y. Tang X. N. Yenari M. A. (2012). NOX inhibitors as a therapeutic strategy for stroke and neurodegenerative disease. *Curr. Drug Targets* 13 199–206. 10.2174/138945012799201676 22204319

[B14] Chalupsky K. Cai H. (2005). Endothelial dihydrofolate reductase: Critical for nitric oxide bioavailability and role in angiotensin II uncoupling of endothelial nitric oxide synthase. *Proc. Natl. Acad. Sci. U.S.A.* 102 9056–9061. 10.1073/pnas.0409594102 15941833 PMC1157015

[B15] Cheng G. Cao Z. Xu X. Van Meir E. G. Lambeth J. D. (2001). Homologs of gp91phox: Cloning and tissue expression of Nox3, Nox4, and Nox5. *Gene* 269 131–140. 10.1016/S0378-1119(01)00449-8 11376945

[B16] Chrissobolis S. Banfi B. Sobey C. G. Faraci F. M. (2012). Role of Nox isoforms in angiotensin II-induced oxidative stress and endothelial dysfunction in brain. *J. Appl. Physiol.* 113 184–191. 10.1152/japplphysiol.00455.2012 22628375 PMC3774474

[B17] Clarke J. V. Suggs J. M. Diwan D. Lee J. V. Lipsey K. Vellimana A. K. (2020). Microvascular platelet aggregation and thrombosis after subarachnoid hemorrhage: A review and synthesis. *J. Cereb. Blood Flow Metab.* 40 1565–1575. 10.1177/0271678X20921974 32345104 PMC7370365

[B18] Deffieux T. Demene C. Pernot M. Tanter M. (2018). Functional ultrasound neuroimaging: A review of the preclinical and clinical state of the art. *Curr. Opin. Neurobiol.* 50 128–135. 10.1016/j.conb.2018.02.001 29477979

[B19] Deng H. J. Deji Q. Zhaba W. Liu J. Q. Gao S. Q. Han Y. L. (2021). A20 establishes negative feedback with TRAF6/NF-κB and attenuates early brain injury after experimental subarachnoid hemorrhage. *Front. Immunol.* 12:623256. 10.3389/fimmu.2021.623256 34381441 PMC8350325

[B20] Di Wang H. Hope S. Du Y. Quinn M. T. Cayatte A. Pagano P. J. (1999). Paracrine role of adventitial superoxide anion in mediating spontaneous tone of the isolated rat aorta in angiotensin II-induced hypertension. *Hypertension* 33 1225–1232. 10.1161/01.HYP.33.5.1225 10334816

[B21] DiNapoli V. A. Huber J. D. Houser K. Li X. Rosen C. L. (2008). Early disruptions of the blood-brain barrier may contribute to exacerbated neuronal damage and prolonged functional recovery following stroke in aged rats. *Neurobiol. Aging* 29 753–764. 10.1016/j.neurobiolaging.2006.12.007 17241702 PMC2683361

[B22] Dunn A. K. (2012). Laser speckle contrast imaging of cerebral blood flow. *Ann. Biomed. Eng.* 40 367–377. 10.1007/s10439-011-0469-0 22109805 PMC3288249

[B23] Fan J. L. Nogueira R. C. Brassard P. Rickards C. A. Page M. Nasr N. (2022). Integrative physiological assessment of cerebral hemodynamics and metabolism in acute ischemic stroke. *J. Cereb. Blood Flow Metab.* 42 454–470. 10.1177/0271678X211033732 34304623 PMC8985442

[B24] Förstermann U. (2008). Oxidative stress in vascular disease: Causes, defense mechanisms and potential therapies. *Nat. Clin. Pract. Cardiovasc. Med.* 5 338–349. 10.1038/ncpcardio1211 18461048

[B25] Förstermann U. (2010). Nitric oxide and oxidative stress in vascular disease. *Pflugers Arch.* 459 923–939. 10.1007/s00424-010-0808-2 20306272

[B26] Förstermann U. Münzel T. (2006). Endothelial nitric oxide synthase in vascular disease: From marvel to menace. *Circulation* 113 1708–1714. 10.1161/CIRCULATIONAHA.105.602532 16585403

[B27] Förstermann U. Sessa W. C. (2012). Nitric oxide synthases: Regulation and function. *Eur. Heart J.* 33 29–37, 837a–837d. 10.1093/eurheartj/ehr304 21890489 PMC3345541

[B28] Friedrich B. Müller F. Feiler S. Schöller K. Plesnila N. (2012). Experimental subarachnoid hemorrhage causes early and long-lasting microarterial constriction and microthrombosis: An in-vivo microscopy study. *J. Cereb. Blood Flow Metab.* 32 447–455. 10.1038/jcbfm.2011.154 22146194 PMC3293113

[B29] Fuloria S. Subramaniyan V. Karupiah S. Kumari U. Sathasivam K. Meenakshi D. U. (2021). Comprehensive review of methodology to detect reactive oxygen species (ROS) in mammalian species and establish its relationship with antioxidants and cancer. *Antioxidants* 10:128. 10.3390/antiox10010128 33477494 PMC7831054

[B30] Gao L. Chalupsky K. Stefani E. Cai H. (2009). Mechanistic insights into folic acid-dependent vascular protection: Dihydrofolate reductase (DHFR)-mediated reduction in oxidant stress in endothelial cells and angiotensin II-infused mice: A novel HPLC-based fluorescent assay for DHFR activity. *J. Mol. Cell Cardiol.* 47 752–760. 10.1016/j.yjmcc.2009.07.025 19660467 PMC2784291

[B31] Gao L. Siu K. L. Chalupsky K. Nguyen A. Chen P. Weintraub N. L. (2012). Role of uncoupled endothelial nitric oxide synthase in abdominal aortic aneurysm formation: Treatment with folic acid. *Hypertension* 59 158–166. 10.1161/HYPERTENSIONAHA.111.181644 22083158 PMC3668799

[B32] Gao S. Q. Liu J. Q. Han Y. L. Deji Q. Z. Zhaba W. D. Deng H. J. (2020). Neuroprotective role of glutathione peroxidase 4 in experimental subarachnoid hemorrhage models. *Life Sci.* 257:118050. 10.1016/j.lfs.2020.118050 32634425

[B33] Gao S. Q. Shi J. J. Xue W. Miao S. H. Li T. Gao C. C. (2022). Endothelial NOX4 aggravates eNOS uncoupling by decreasing dihydrofolate reductase after subarachnoid hemorrhage. *Free Radic. Biol. Med.* 193 499–510. 10.1016/j.freeradbiomed.2022.10.318 36336227

[B34] Gordon G. R. Howarth C. Macvicar B. A. (2011). Bidirectional control of arteriole diameter by astrocytes. *Exp. Physiol.* 96 393–399. 10.1113/expphysiol.2010.053132 21257665

[B35] Grote E. Hassler W. (1988). The critical first minutes after subarachnoid hemorrhage. *Neurosurgery* 22 654–661. 10.1227/00006123-198804000-00006 3287211

[B36] Hansen-Schwartz J. Hoel N. L. Zhou M. Xu C. B. Svendgaard N. A. Edvinsson L. (2003). Subarachnoid hemorrhage enhances endothelin receptor expression and function in rat cerebral arteries. *Neurosurgery* 52 1188–1194; 1194–1195. 10.1227/01.NEU.0000058467.82442.64 12699564

[B37] Hop J. W. Rinkel G. J. Algra A. Van Gijn J. (1997). Case-fatality rates and functional outcome after subarachnoid hemorrhage: A systematic review. *Stroke* 28 660–664. 10.1161/01.STR.28.3.660 9056628

[B38] Horky L. L. Pluta R. M. Boock R. J. Oldfield E. H. (1998). Role of ferrous iron chelator 2,2’-dipyridyl in preventing delayed vasospasm in a primate model of subarachnoid hemorrhage. *J. Neurosurg.* 88 298–303. 10.3171/jns.1998.88.2.0298 9452239

[B39] Iadecola C. Nedergaard M. (2007). Glial regulation of the cerebral microvasculature. *Nat. Neurosci.* 10 1369–1376. 10.1038/nn2003 17965657

[B40] Iadecola C. Park L. Capone C. (2009). Threats to the mind: Aging, amyloid, and hypertension. *Stroke* 40 S40–S44. 10.1161/STROKEAHA.108.533638 19064785 PMC2704500

[B41] Jung O. Marklund S. L. Geiger H. Pedrazzini T. Busse R. Brandes R. P. (2003). Extracellular superoxide dismutase is a major determinant of nitric oxide bioavailability: In vivo and ex vivo evidence from ecSOD-deficient mice. *Circ. Res.* 93 622–629. 10.1161/01.RES.0000092140.81594.A8 12933702

[B42] Lambeth J. D. Neish A. S. (2014). Nox enzymes and new thinking on reactive oxygen: A double-edged sword revisited. *Annu. Rev. Pathol.* 9 119–145. 10.1146/annurev-pathol-012513-104651 24050626

[B43] Landmesser U. Dikalov S. Price S. R. Mccann L. Fukai T. Holland S. M. (2003). Oxidation of tetrahydrobiopterin leads to uncoupling of endothelial cell nitric oxide synthase in hypertension. *J. Clin. Invest.* 111 1201–1209. 10.1172/JCI200314172 12697739 PMC152929

[B44] Laursen J. B. Somers M. Kurz S. Mccann L. Warnholtz A. Freeman B. A. (2001). Endothelial regulation of vasomotion in apoE-deficient mice: Implications for interactions between peroxynitrite and tetrahydrobiopterin. *Circulation* 103 1282–1288. 10.1161/01.CIR.103.9.1282 11238274

[B45] Lenz I. J. Plesnila N. Terpolilli N. A. (2021). Role of endothelial nitric oxide synthase for early brain injury after subarachnoid hemorrhage in mice. *J. Cereb. Blood Flow Metab.* 41 1669–1681. 10.1177/0271678X20973787 33256507 PMC8221759

[B46] Li H. Li Q. Zhang Y. Liu W. Gu B. Narumi T. (2019). Novel treatment of hypertension by specifically targeting E2F for restoration of endothelial dihydrofolate reductase and eNOS function under oxidative stress. *Hypertension* 73 179–189. 10.1161/HYPERTENSIONAHA.118.11643 30571557 PMC6310047

[B47] Li Q. Youn J. Y. Cai H. (2015). Mechanisms and consequences of endothelial nitric oxide synthase dysfunction in hypertension. *J. Hypertens.* 33 1128–1136. 10.1097/HJH.0000000000000587 25882860 PMC4816601

[B48] Lindskog C. Asplund A. Catrina A. Nielsen S. Rützler M. (2016). A systematic characterization of aquaporin-9 expression in human normal and pathological tissues. *J. Histochem. Cytochem.* 64 287–300. 10.1369/0022155416641028 27026296 PMC4851273

[B49] Liu Z. Chopp M. (2016). Astrocytes, therapeutic targets for neuroprotection and neurorestoration in ischemic stroke. *Prog. Neurobiol.* 144 103–120. 10.1016/j.pneurobio.2015.09.008 26455456 PMC4826643

[B50] Macdonald R. L. Schweizer T. A. (2017). Spontaneous subarachnoid haemorrhage. *Lancet* 389 655–666. 10.1016/S0140-6736(16)30668-7 27637674

[B51] Macdonald R. L. Weir B. K. (1994). Cerebral vasospasm and free radicals. *Free Radic. Biol. Med.* 16 633–643. 10.1016/0891-5849(94)90064-7 8026807

[B52] Marchetto M. C. Muotri A. R. Mu Y. Smith A. M. Cezar G. G. Gage F. H. (2008). Non-cell-autonomous effect of human SOD1 G37R astrocytes on motor neurons derived from human embryonic stem cells. *Cell Stem Cell* 3 649–657. 10.1016/j.stem.2008.10.001 19041781

[B53] Marklund S. L. (1984). Extracellular superoxide dismutase and other superoxide dismutase isoenzymes in tissues from nine mammalian species. *Biochem. J.* 222 649–655. 10.1042/bj2220649 6487268 PMC1144226

[B54] Matz P. G. Copin J. C. Chan P. H. (2000). Cell death after exposure to subarachnoid hemolysate correlates inversely with expression of CuZn-superoxide dismutase. *Stroke* 31 2450–2459. 10.1161/01.STR.31.10.2450 11022079

[B55] Mayberg M. R. Batjer H. H. Dacey R. Diringer M. Haley E. C. Heros R. C. (1994). Guidelines for the management of aneurysmal subarachnoid hemorrhage. A statement for healthcare professionals from a special writing group of the Stroke Council, American Heart Association. *Circulation* 90 2592–2605. 10.1161/01.CIR.90.5.2592 7955232

[B56] McGirt M. J. Parra A. Sheng H. Higuchi Y. Oury T. D. Laskowitz D. T. (2002b). Attenuation of cerebral vasospasm after subarachnoid hemorrhage in mice overexpressing extracellular superoxide dismutase. *Stroke* 33 2317–2323. 10.1161/01.STR.0000027207.67639.1E 12215605

[B57] McGirt M. J. Lynch J. R. Parra A. Sheng H. Pearlstein R. D. Laskowitz D. T. (2002a). Simvastatin increases endothelial nitric oxide synthase and ameliorates cerebral vasospasm resulting from subarachnoid hemorrhage. *Stroke* 33 2950–2956. 10.1161/01.STR.0000038986.68044.39 12468796

[B58] Miller A. A. De Silva T. M. Judkins C. P. Diep H. Drummond G. R. Sobey C. G. (2010). Augmented superoxide production by Nox2-containing NADPH oxidase causes cerebral artery dysfunction during hypercholesterolemia. *Stroke* 41 784–789. 10.1161/STROKEAHA.109.575365 20167907

[B59] Miller A. A. Drummond G. R. Sobey C. G. (2006). Novel isoforms of Nadph-oxidase in cerebral vascular control. *Pharmacol. Ther.* 111 928–948. 10.1016/j.pharmthera.2006.02.005 16616784

[B60] Mir D. I. Gupta A. Dunning A. Puchi L. Robinson C. L. Epstein H. A. (2014). CT perfusion for detection of delayed cerebral ischemia in aneurysmal subarachnoid hemorrhage: A systematic review and meta-analysis. *AJNR Am. J. Neuroradiol.* 35 866–871. 10.3174/ajnr.A3787 24309123 PMC4159608

[B61] Nauseef W. M. (2019). The phagocyte NOX2 NADPH oxidase in microbial killing and cell signaling. *Curr. Opin. Immunol.* 60 130–140. 10.1016/j.coi.2019.05.006 31302569 PMC6800624

[B62] Oak J. H. Cai H. (2007). Attenuation of angiotensin II signaling recouples eNOS and inhibits nonendothelial NOX activity in diabetic mice. *Diabetes* 56 118–126. 10.2337/db06-0288 17192473

[B63] Ostrowski R. P. Colohan A. R. Zhang J. H. (2006b). Neuroprotective effect of hyperbaric oxygen in a rat model of subarachnoid hemorrhage. *Acta Neurochir. Suppl.* 96 188–193. 10.1007/3-211-30714-1_41 16671452

[B64] Ostrowski R. P. Tang J. Zhang J. H. (2006c). Hyperbaric oxygen suppresses NADPH oxidase in a rat subarachnoid hemorrhage model. *Stroke* 37 1314–1318. 10.1161/01.STR.0000217310.88450.c3 16556878

[B65] Ostrowski R. P. Colohan A. R. Zhang J. H. (2006a). Molecular mechanisms of early brain injury after subarachnoid hemorrhage. *Neurol. Res.* 28 399–414. 10.1179/016164106X115008 16759443

[B66] Pappas A. C. Koide M. Wellman G. C. (2015). Astrocyte Ca2+ signaling drives inversion of neurovascular coupling after subarachnoid hemorrhage. *J. Neurosci.* 35 13375–13384. 10.1523/JNEUROSCI.1551-15.2015 26424885 PMC4588610

[B67] Paravicini T. M. Sobey C. G. (2003). Cerebral vascular effects of reactive oxygen species: Recent evidence for a role of NADPH-oxidase. *Clin. Exp. Pharmacol. Physiol.* 30 855–859. 10.1046/j.1440-1681.2003.03920.x 14678250

[B68] Pennings F. A. Bouma G. J. Ince C. (2004). Direct observation of the human cerebral microcirculation during aneurysm surgery reveals increased arteriolar contractility. *Stroke* 35 1284–1288. 10.1161/01.STR.0000126039.91400.cb 15087565

[B69] Plesnila N. (2013). Pathophysiological role of global cerebral ischemia following subarachnoid hemorrhage: The current experimental evidence. *Stroke Res. Treat.* 2013:651958. 10.1155/2013/651958 23844316 PMC3694494

[B70] Rao G. S. Durga P. (2011). Changing trends in monitoring brain ischemia: From intracranial pressure to cerebral oximetry. *Curr. Opin. Anaesthesiol.* 24 487–494. 10.1097/ACO.0b013e32834a8965 21799403

[B71] Rey F. E. Li X. C. Carretero O. A. Garvin J. L. Pagano P. J. (2002). Perivascular superoxide anion contributes to impairment of endothelium-dependent relaxation: Role of gp91(phox). *Circulation* 106 2497–2502. 10.1161/01.CIR.0000038108.71560.70 12417549

[B72] Sabri M. Ai J. Knight B. Tariq A. Jeon H. Shang X. (2011a). Uncoupling of endothelial nitric oxide synthase after experimental subarachnoid hemorrhage. *J. Cereb. Blood Flow Metab.* 31 190–199. 10.1038/jcbfm.2010.76 20517322 PMC3049483

[B73] Sabri M. Ai J. Marsden P. A. Macdonald R. L. (2011b). Simvastatin re-couples dysfunctional endothelial nitric oxide synthase in experimental subarachnoid hemorrhage. *PLoS One* 6:e17062. 10.1371/journal.pone.0017062 21373645 PMC3044158

[B74] Sabri M. Ai J. Lakovic K. D’abbondanza J. Ilodigwe D. Macdonald R. L. (2012). Mechanisms of microthrombi formation after experimental subarachnoid hemorrhage. *Neuroscience* 224 26–37. 10.1016/j.neuroscience.2012.08.002 22902542

[B75] Sabri M. Jeon H. Ai J. Tariq A. Shang X. Chen G. (2009). Anterior circulation mouse model of subarachnoid hemorrhage. *Brain Res.* 1295 179–185. 10.1016/j.brainres.2009.08.021 19686712

[B76] Schubert G. A. Seiz M. Hegewald A. A. Manville J. Thomé C. (2009). Acute hypoperfusion immediately after subarachnoid hemorrhage: A xenon contrast-enhanced CT study. *J. Neurotrauma* 26 2225–2231. 10.1089/neu.2009.0924 19929373

[B77] Schwartz A. Y. Sehba F. A. Bederson J. B. (2000). Decreased nitric oxide availability contributes to acute cerebral ischemia after subarachnoid hemorrhage. *Neurosurgery* 47 208–214; discussion 214–215. 10.1227/00006123-200007000-00042 10917364

[B78] Sehba F. A. Ding W. H. Chereshnev I. Bederson J. B. (1999). Effects of S-nitrosoglutathione on acute vasoconstriction and glutamate release after subarachnoid hemorrhage. *Stroke* 30 1955–1961. 10.1161/01.STR.30.9.1955 10471450

[B79] Sehba F. A. Hou J. Pluta R. M. Zhang J. H. (2012). The importance of early brain injury after subarachnoid hemorrhage. *Prog. Neurobiol.* 97 14–37. 10.1016/j.pneurobio.2012.02.003 22414893 PMC3327829

[B80] Sehba F. A. Schwartz A. Y. Chereshnev I. Bederson J. B. (2000). Acute decrease in cerebral nitric oxide levels after subarachnoid hemorrhage. *J. Cereb. Blood Flow Metab.* 20 604–611. 10.1097/00004647-200003000-00018 10724124

[B81] Shin H. K. Lee J. H. Kim K. Y. Kim C. D. Lee W. S. Rhim B. Y. (2002). Impairment of autoregulatory vasodilation by NAD(P)H oxidase-dependent superoxide generation during acute stage of subarachnoid hemorrhage in rat pial artery. *J. Cereb. Blood Flow Metab.* 22 869–877. 10.1097/00004647-200207000-00012 12142572

[B82] Sun B. L. Zheng C. B. Yang M. F. Yuan H. Zhang S. M. Wang L. X. (2009). Dynamic alterations of cerebral pial microcirculation during experimental subarachnoid hemorrhage. *Cell. Mol. Neurobiol.* 29 235–241. 10.1007/s10571-008-9316-8 18821009 PMC11505794

[B83] Terpolilli N. A. Brem C. Bühler D. Plesnila N. (2015). Are we barking up the wrong vessels? Cerebral microcirculation after subarachnoid hemorrhage. *Stroke* 46 3014–3019. 10.1161/STROKEAHA.115.006353 26152299

[B84] Terpolilli N. A. Feiler S. Dienel A. Müller F. Heumos N. Friedrich B. (2016). Nitric oxide inhalation reduces brain damage, prevents mortality, and improves neurological outcome after subarachnoid hemorrhage by resolving early pial microvasospasms. *J. Cereb. Blood Flow Metab.* 36 2096–2107. 10.1177/0271678X15605848 26661144 PMC5363657

[B85] Thannickal V. J. Fanburg B. L. (2000). Reactive oxygen species in cell signaling. *Am. J. Physiol. Lung Cell. Mol. Physiol.* 279:L1005-28. 10.1152/ajplung.2000.279.6.L1005 11076791

[B86] Thannickal V. J. Day R. M. Klinz S. G. Bastien M. C. Larios J. M. Fanburg B. L. (2000). Ras-dependent and -independent regulation of reactive oxygen species by mitogenic growth factors and Tgf-beta1. *FASEB J.* 14 1741–1748. 10.1096/fj.99-0878com 10973923

[B87] Thöny B. Auerbach G. Blau N. (2000). Tetrahydrobiopterin biosynthesis, regeneration and functions. *Biochem. J.* 347(Pt 1) 1–16. 10.1042/bj3470001 10727395 PMC1220924

[B88] Uhl E. Lehmberg J. Steiger H. J. Messmer K. (2003). Intraoperative detection of early microvasospasm in patients with subarachnoid hemorrhage by using orthogonal polarization spectral imaging. *Neurosurgery* 52 1307–1315; discussion 1315–1317. 10.1227/01.NEU.0000065154.04824.9E 12762876

[B89] Waghray M. Cui Z. Horowitz J. C. Subramanian I. M. Martinez F. J. Toews G. B. (2005). Hydrogen peroxide is a diffusible paracrine signal for the induction of epithelial cell death by activated myofibroblasts. *FASEB J.* 19 854–856. 10.1096/fj.04-2882fje 15857893

[B90] Wang H. D. Pagano P. J. Du Y. Cayatte A. J. Quinn M. T. Brecher P. (1998). Superoxide anion from the adventitia of the rat thoracic aorta inactivates nitric oxide. *Circ. Res.* 82 810–818. 10.1161/01.RES.82.7.810 9562441

[B91] Wang X. Li Y. M. Li W. Q. Huang C. G. Lu Y. C. Hou L. J. (2012). Effect of clazosentan in patients with aneurysmal subarachnoid hemorrhage: A meta-analysis of randomized controlled trials. *PLoS One* 7:e47778. 10.1371/journal.pone.0047778 23082215 PMC3474756

[B92] Weksler B. Romero I. A. Couraud P. O. (2013). The hcmec/D3 cell line as a model of the human blood brain barrier. *Fluids Barriers CNS* 10:16. 10.1186/2045-8118-10-16 23531482 PMC3623852

[B93] Winterbourn C. C. (2018). Biological production, detection, and fate of hydrogen peroxide. *Antioxid. Redox Signal.* 29 541–551. 10.1089/ars.2017.7425 29113458

[B94] Xia Y. Tsai A. L. Berka V. Zweier J. L. (1998). Superoxide generation from endothelial nitric-oxide synthase. A Ca2+/calmodulin-dependent and tetrahydrobiopterin regulatory process. *J. Biol. Chem.* 273 25804–25808. 10.1074/jbc.273.40.25804 9748253

[B95] Yang X. M. Chen X. H. Lu J. F. Zhou C. M. Han J. Y. Chen C. H. (2018). In vivo observation of cerebral microcirculation after experimental subarachnoid hemorrhage in mice. *Neural Regen. Res.* 13 456–462. 10.4103/1673-5374.228728 29623930 PMC5900508

[B96] Youn J. Y. Gao L. Cai H. (2012). The p47phox- and Nadph oxidase organiser 1 (NOXO1)-dependent activation of Nadph oxidase 1 (Nox1) mediates endothelial nitric oxide synthase (eNOS) uncoupling and endothelial dysfunction in a streptozotocin-induced murine model of diabetes. *Diabetologia* 55 2069–2079. 10.1007/s00125-012-2557-6 22549734 PMC3694990

[B97] Zhang L. Li Z. Feng D. Shen H. Tian X. Li H. (2017). Involvement of Nox2 and Nox4 NADPH oxidases in early brain injury after subarachnoid hemorrhage. *Free Radic. Res.* 51 316–328. 10.1080/10715762.2017.1311015 28330417

