# Supplementary material for: Increased NOX2 expression in astrocytes leads to eNOS uncoupling through dihydrofolate reductase in endothelial cells after subarachnoid hemorrhage
Source: Front Mol Neurosci. 2023 Mar 30;16:1121944. doi: 10.3389/fnmol.2023.1121944 (PMC10097896; doi:10.3389/fnmol.2023.1121944)

Figure1E

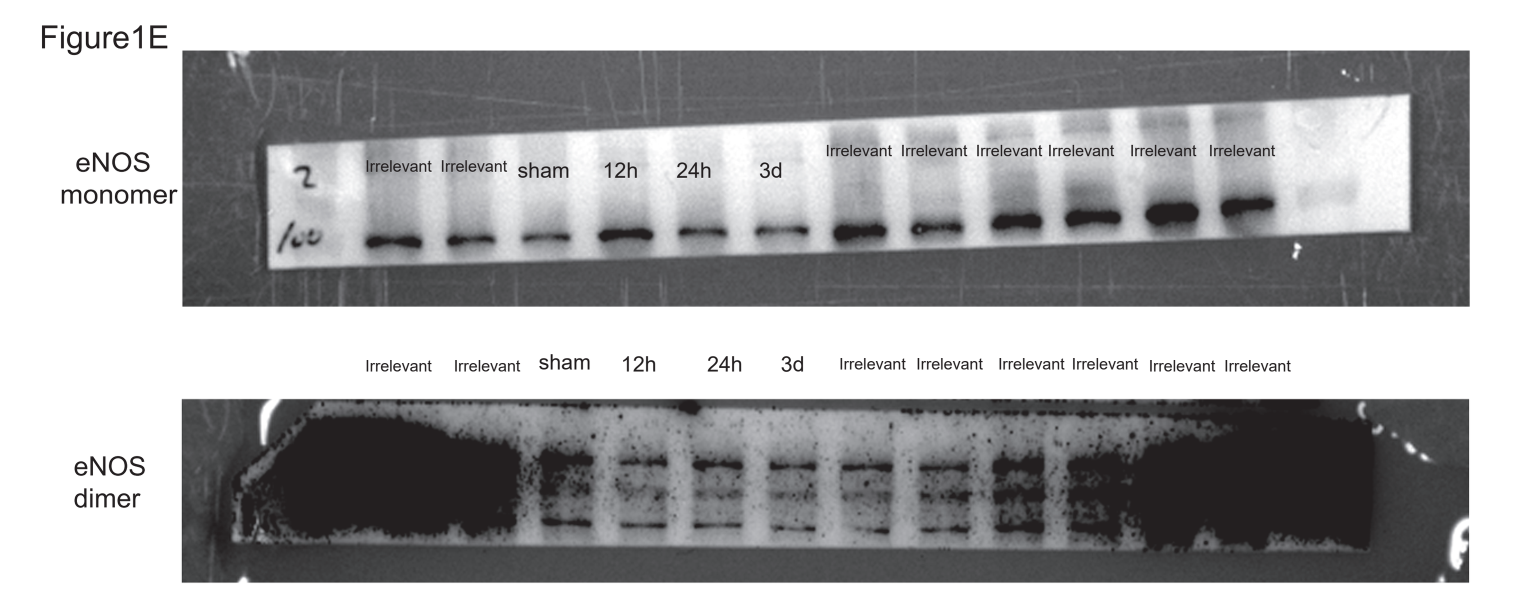

Figure1G

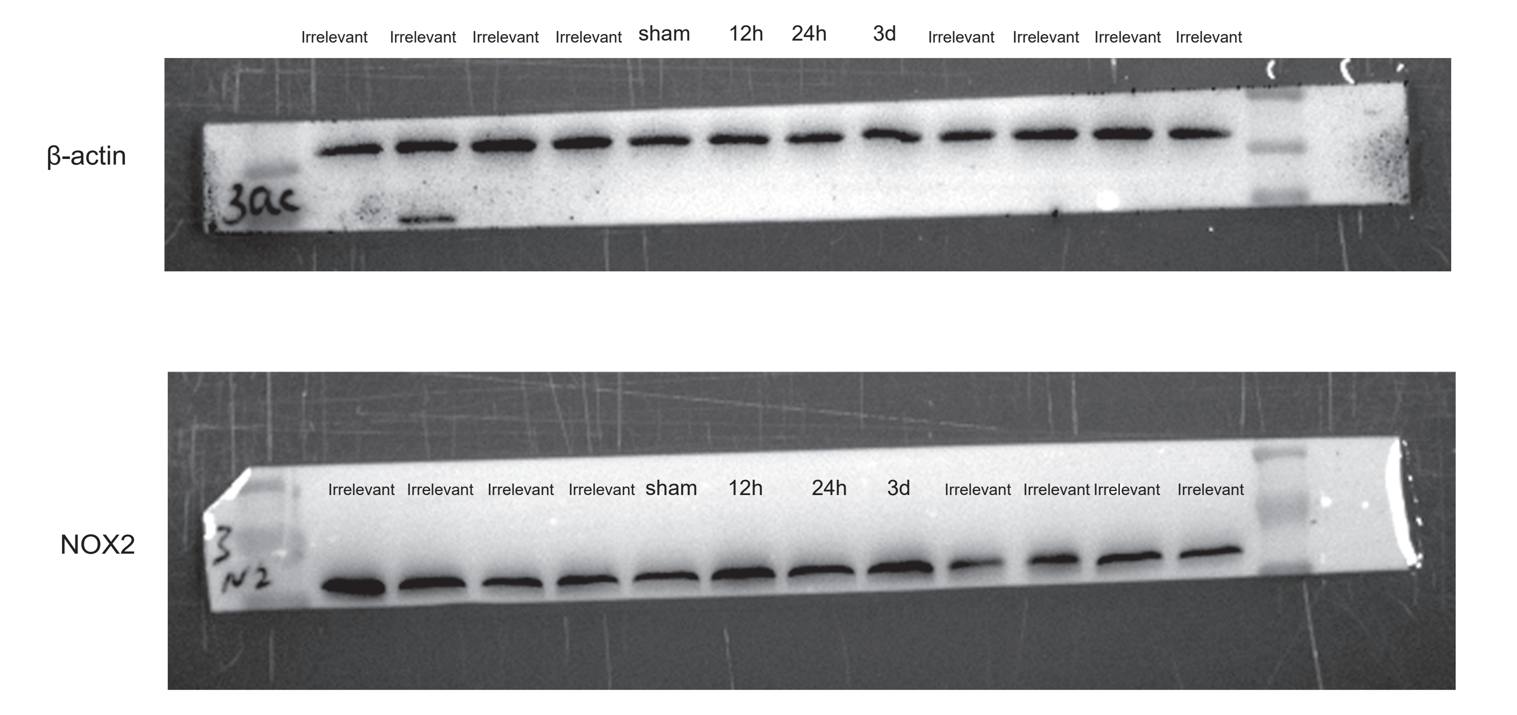

Figure1I

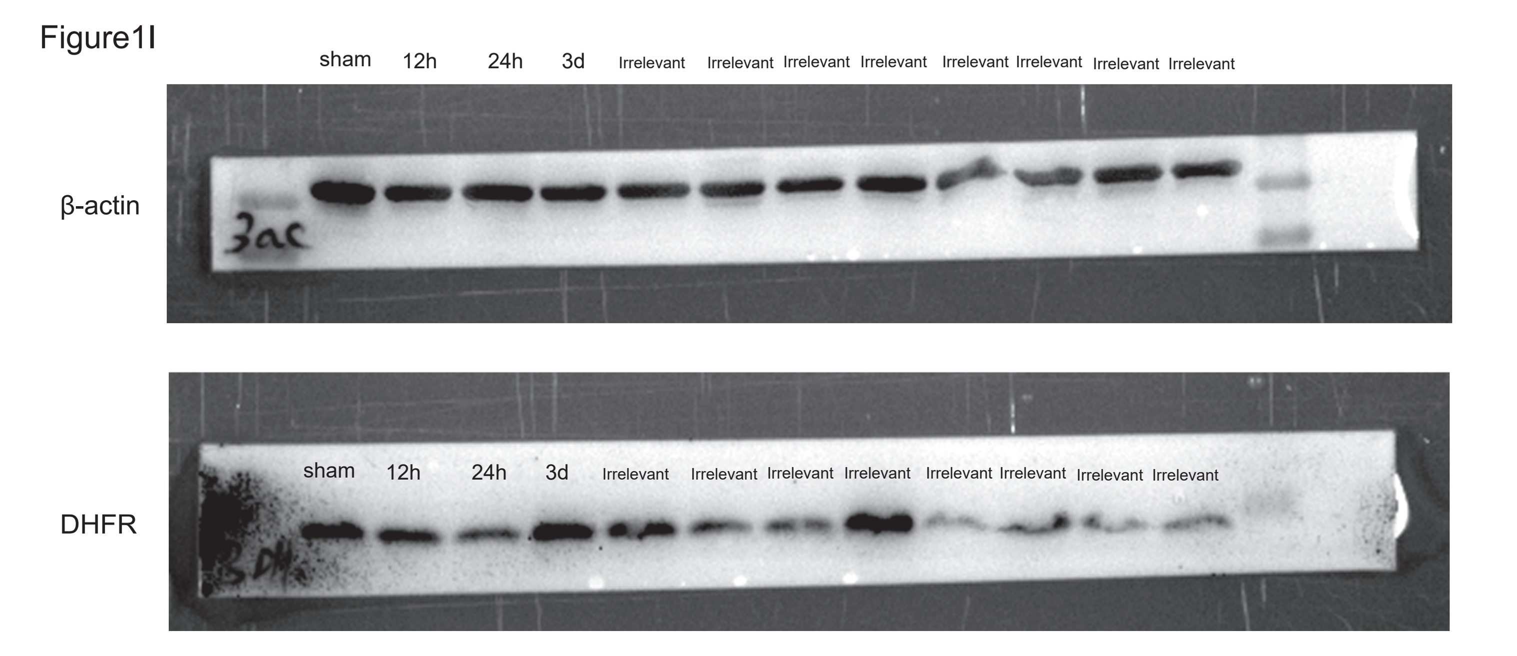

Figure2C

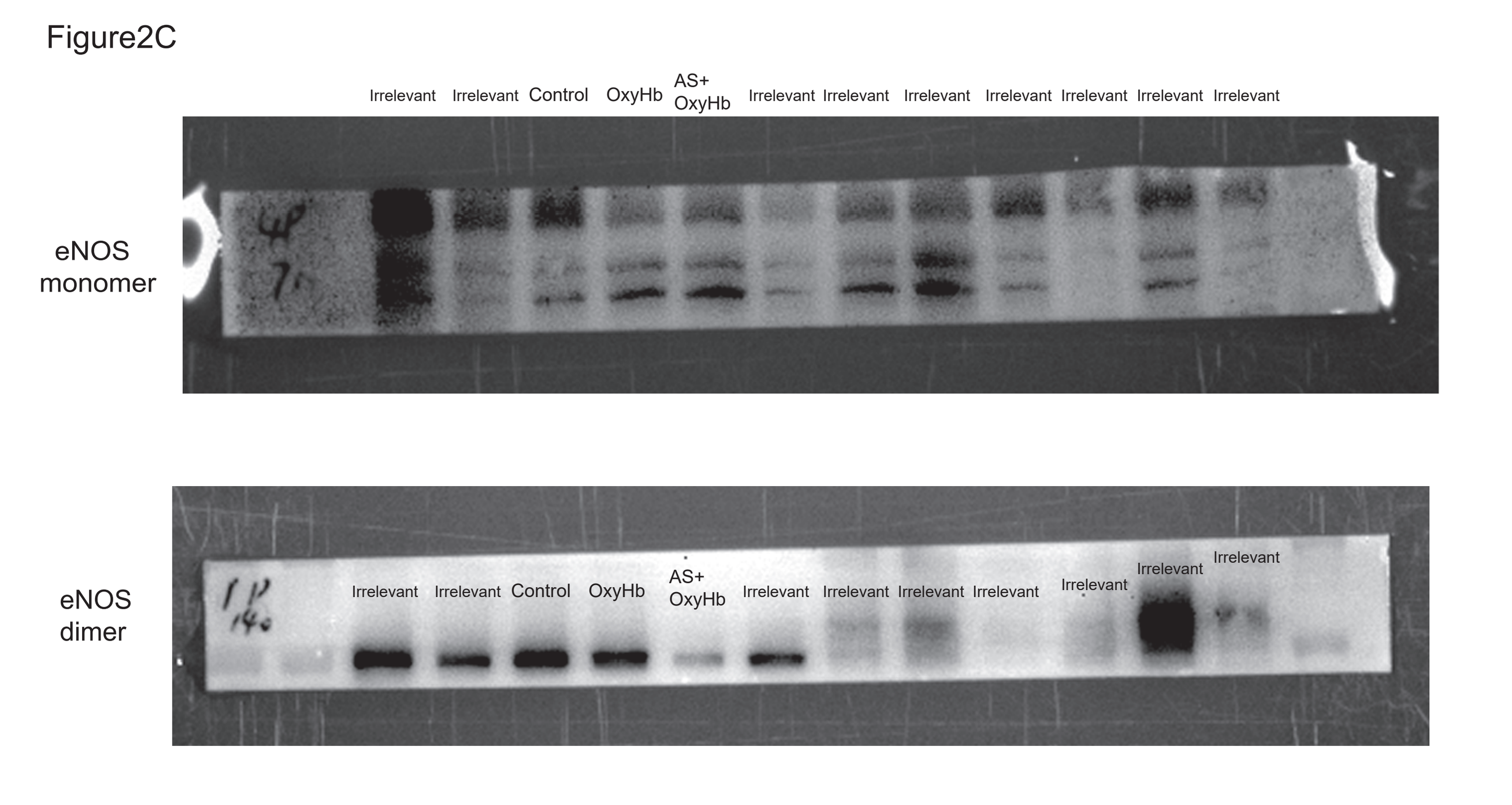

Figure2D

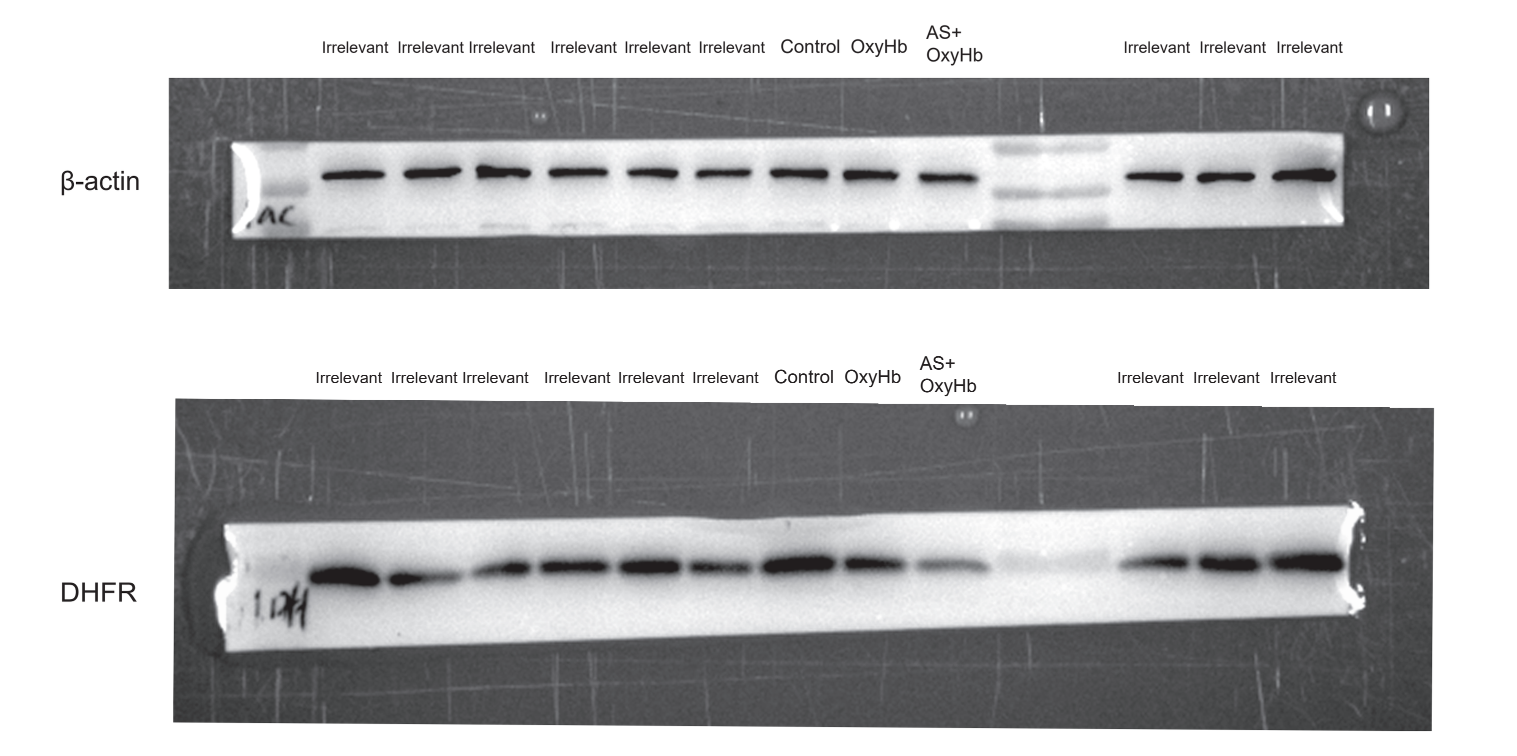

Figure2H

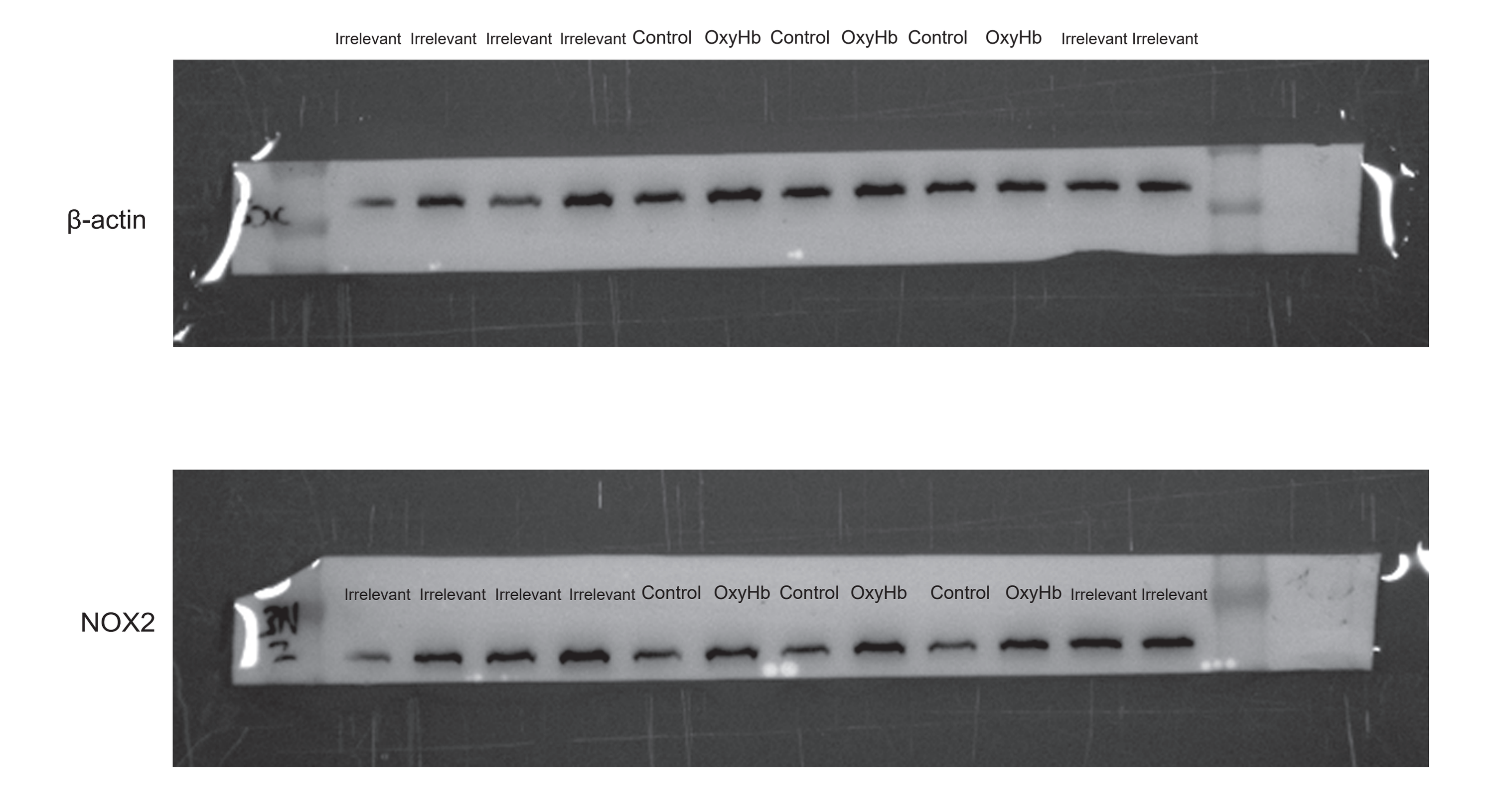

Figure3B

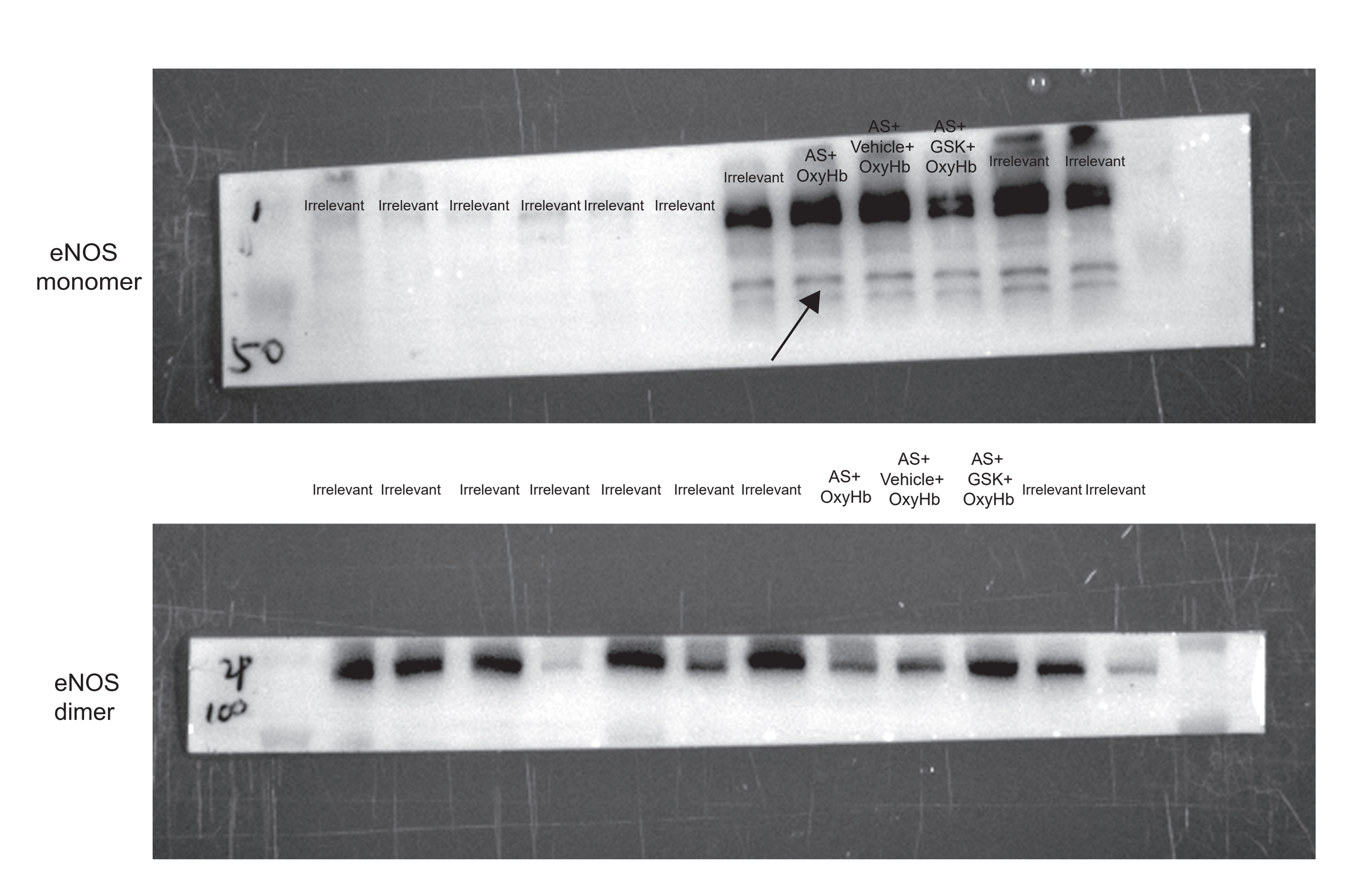

Figure3D

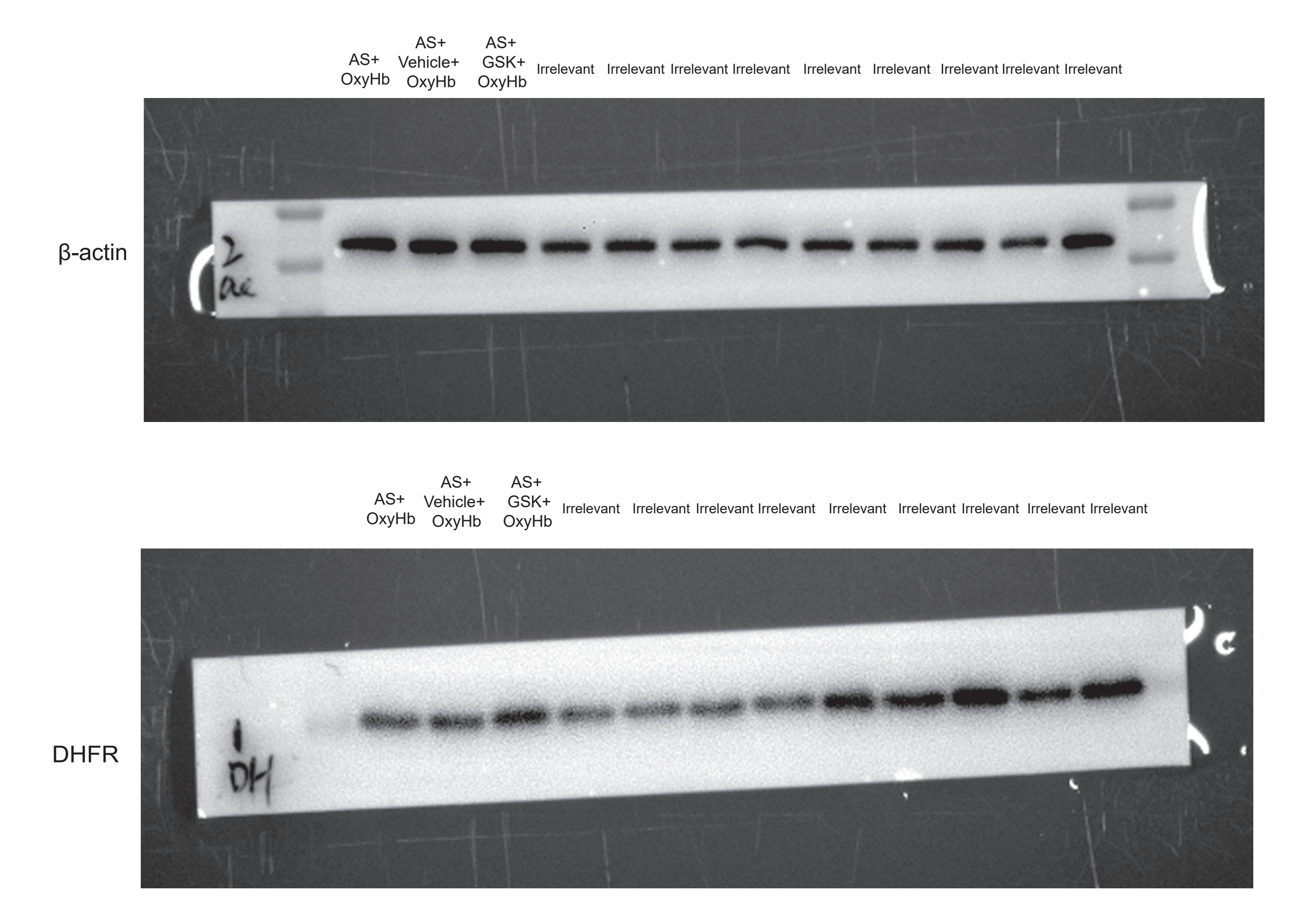

Figure3G

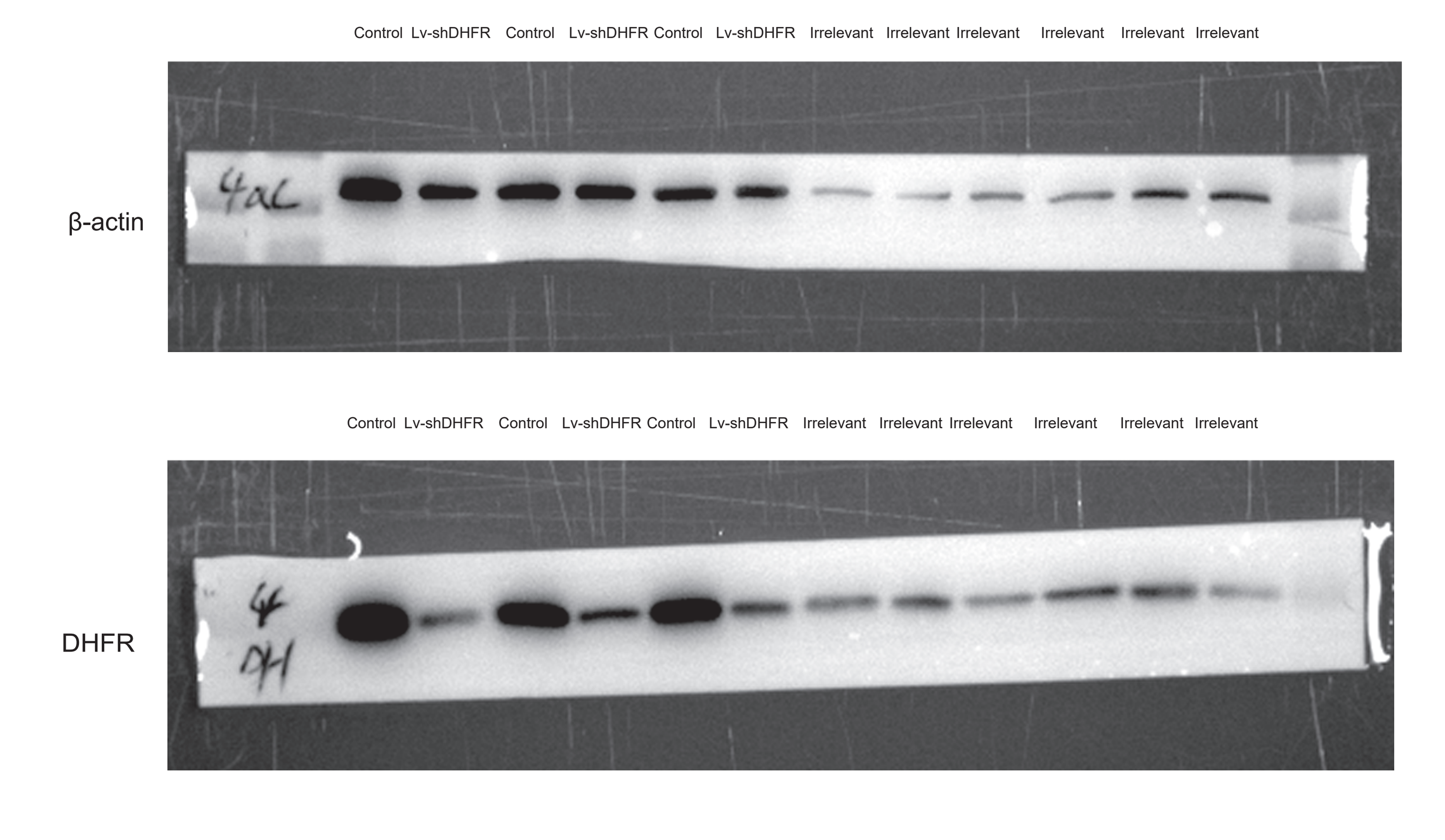

Figure3I

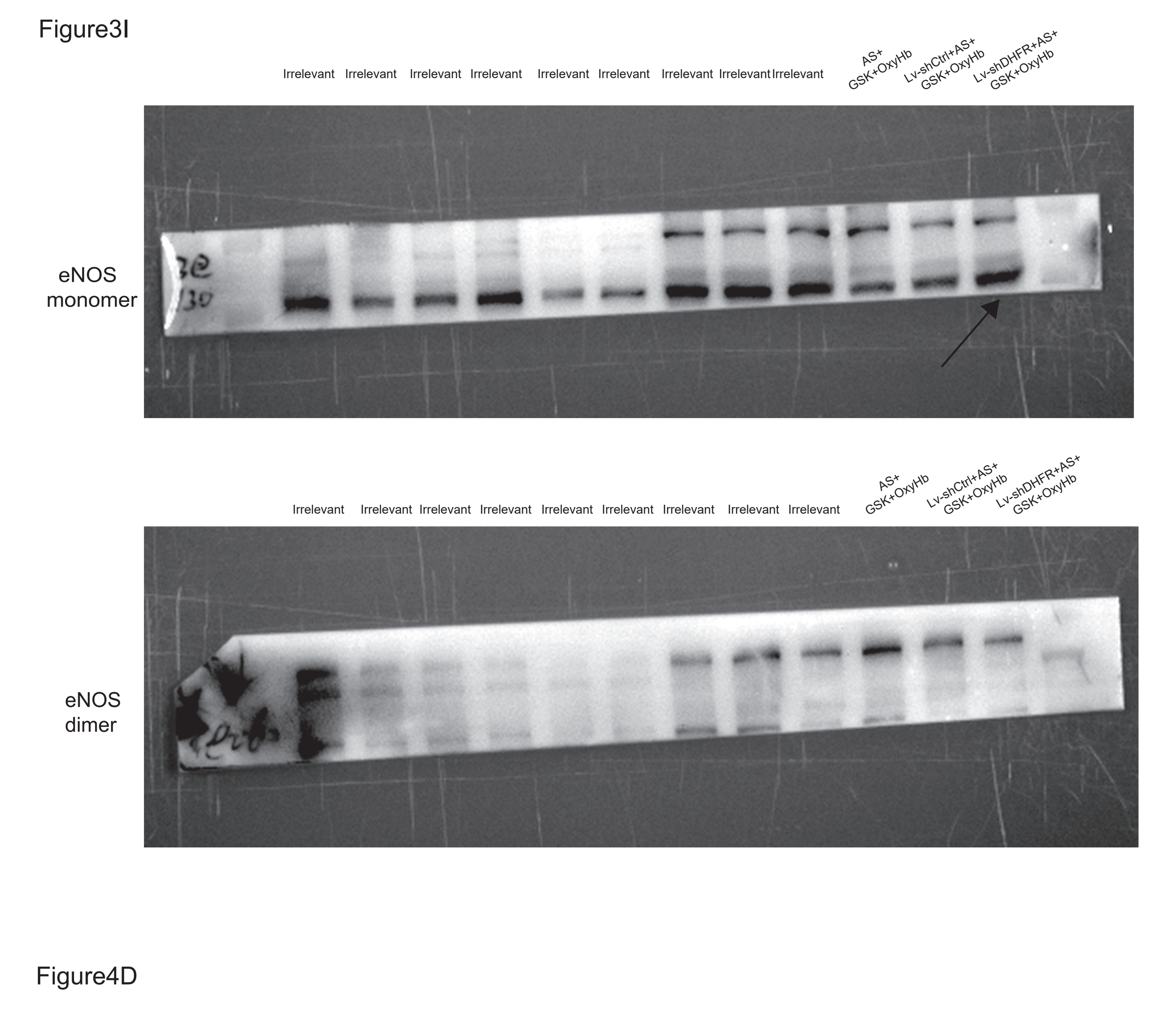

Figure4D

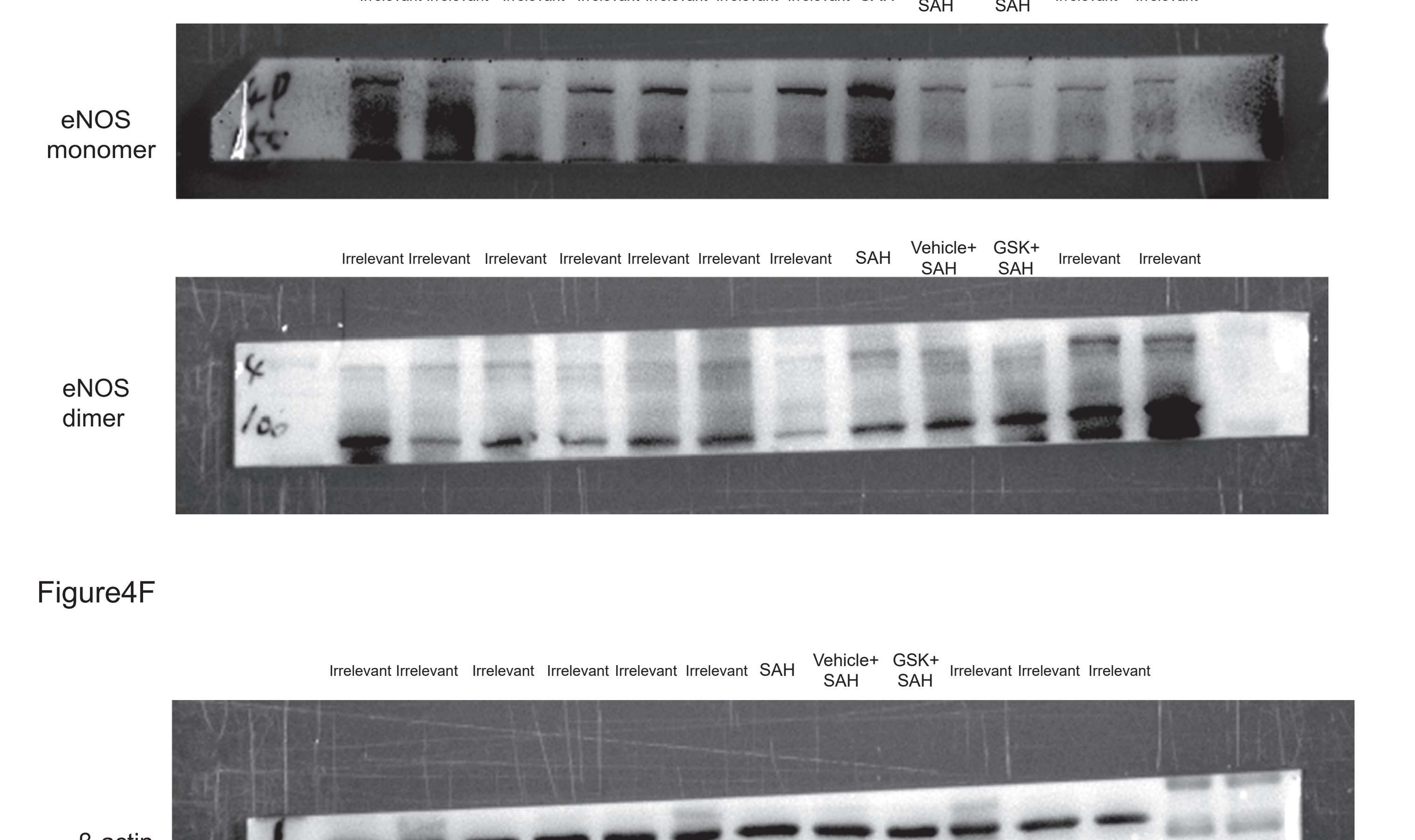

Figure4F

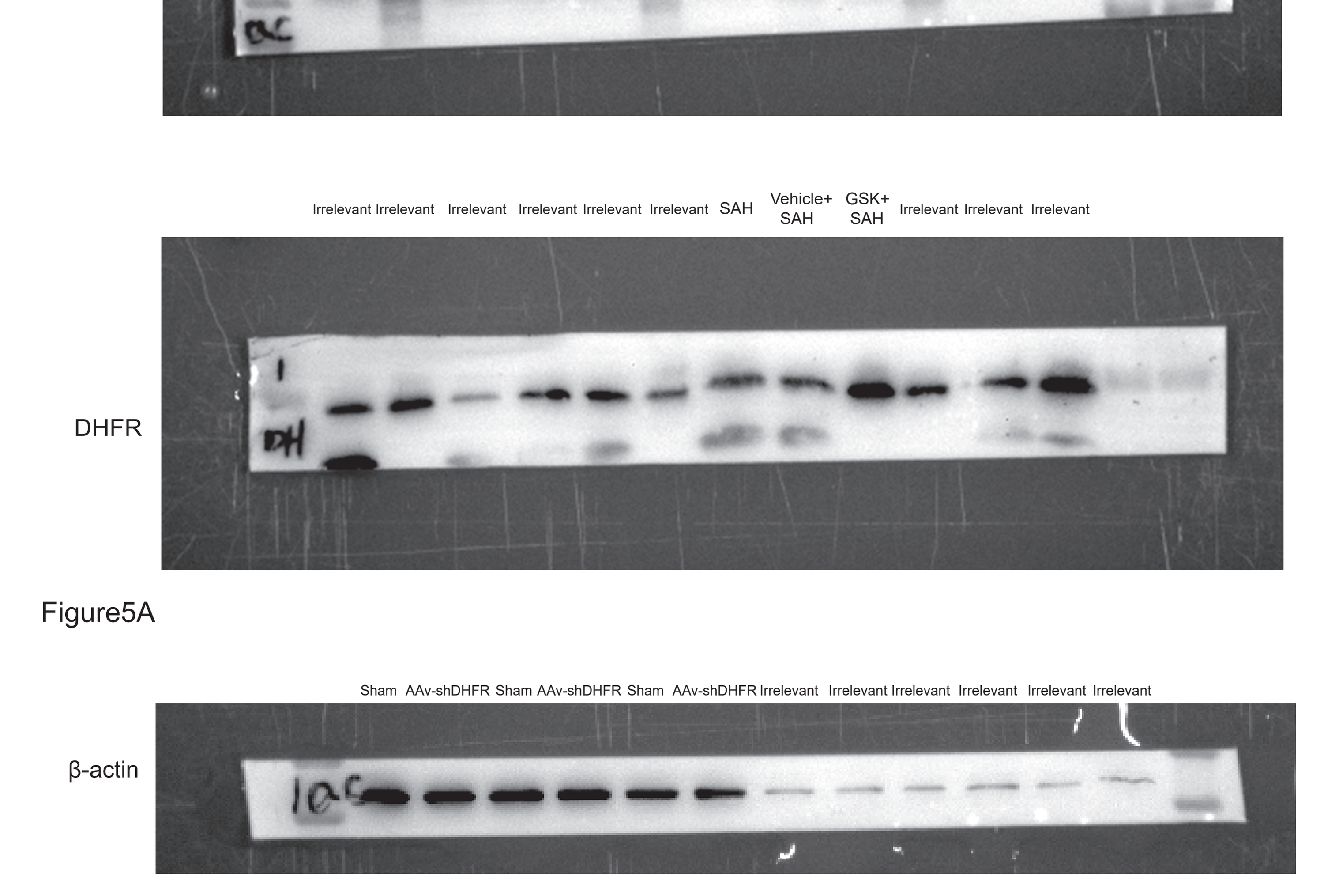

Figure5A

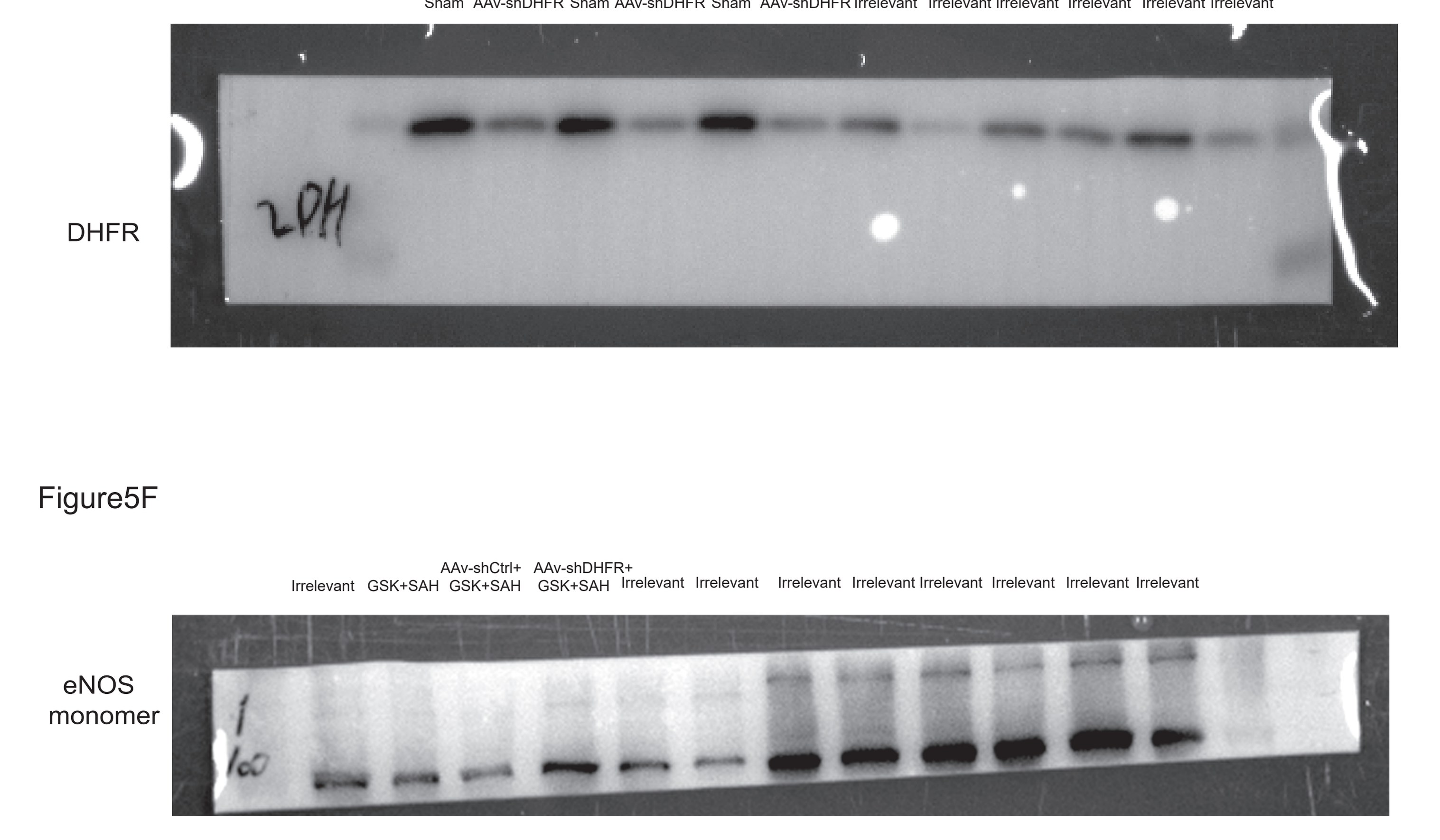

Figure5F

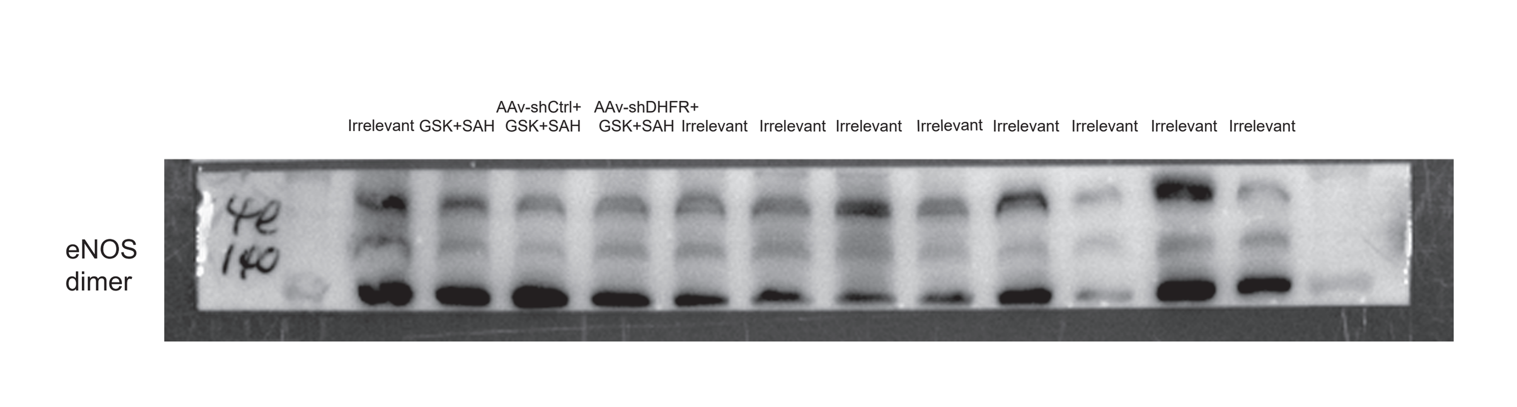

Supplement: Supplementary file 3 [file Data_Sheet_3.pdf]
