# Supplementary material for: Increased NOX2 expression in astrocytes leads to eNOS uncoupling through dihydrofolate reductase in endothelial cells after subarachnoid hemorrhage
Source: Front Mol Neurosci. 2023 Mar 30;16:1121944. doi: 10.3389/fnmol.2023.1121944 (PMC10097896; doi:10.3389/fnmol.2023.1121944)
Supplement: Supplementary file 4 [file Table_1.docx]

| **Group** | **Total** | **Number of dead mice** | **Number of surviving mice** | **Death rate** |
| --- | --- | --- | --- | --- |
| **12h** | **45** | **7** | **38** | **15.56%** |
| **24h** | **30** | **5** | **25** | **16.67%** |
| **3d** | **30** | **6** | **24** | **20%** |
| **SAH** | **45** | **6** | **39** | **13.33%** |
| **Vehicle + SAH** | **45** | **7** | **38** | **15.56%** |
| **GSK + SAH (Figure 4)** | **45** | **4** | **41** | **8.89%** |
| **AAV-shDHFR** | **10** | **0** | **10** | **0%** |
| **GSK + SAH (Figure 5)** | **30** | **3** | **27** | **10%** |
| **AAV-shCtrl + GSK + SAH** | **30** | **3** | **27** | **10%** |
| **AAV-shDHFR + GSK + SAH** | **35** | **9** | **26** | **25.71%** |

**Table S1. *In vivo* experiments, the death rate of mice in each group.**
