# Supplementary figures and images for: Increased NOX2 expression in astrocytes leads to eNOS uncoupling through dihydrofolate reductase in endothelial cells after subarachnoid hemorrhage
Source: Front Mol Neurosci. 2023 Mar 30;16:1121944. doi: 10.3389/fnmol.2023.1121944 (PMC10097896; doi:10.3389/fnmol.2023.1121944)

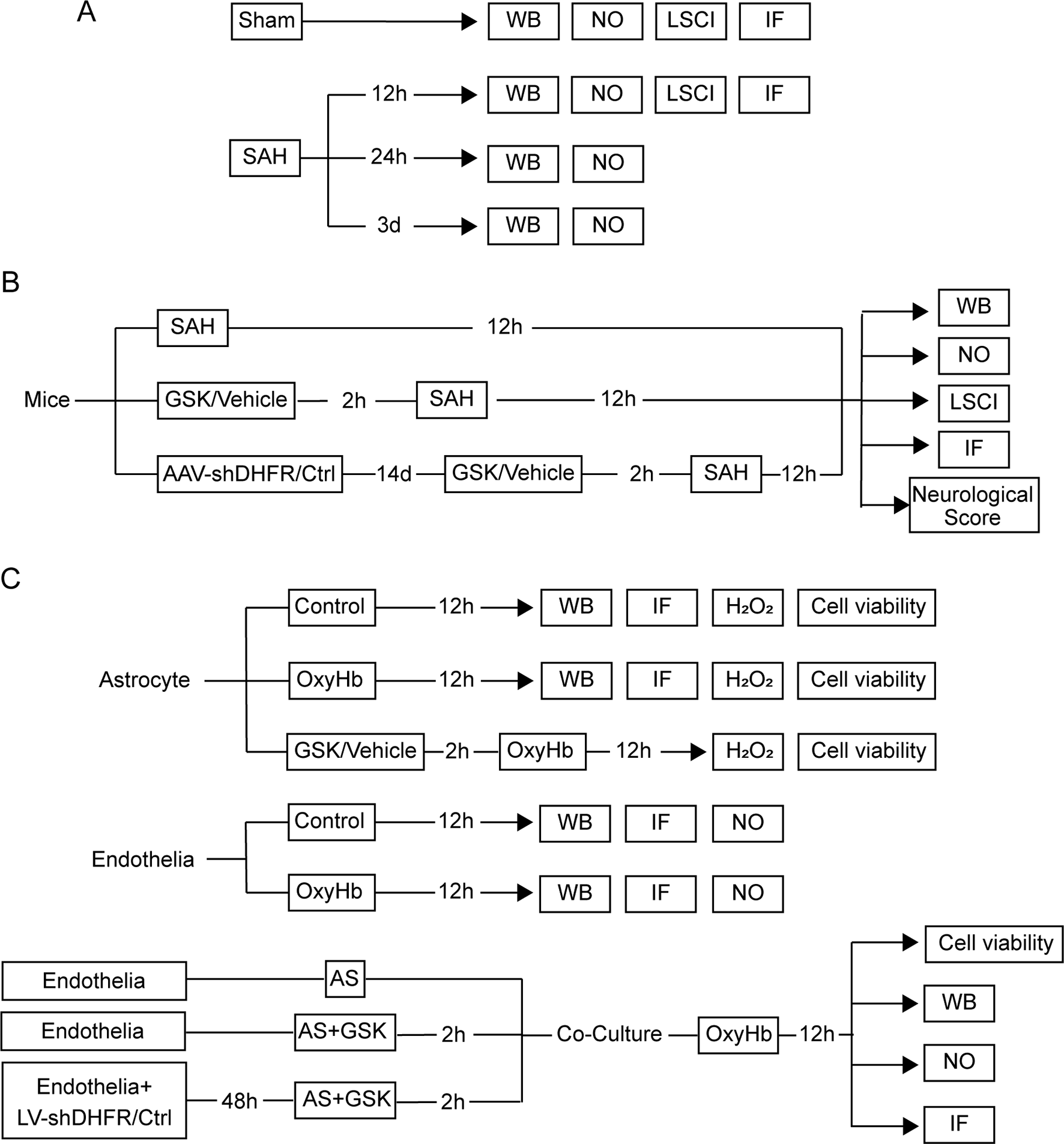

Supplement: Supplementary Figure 1 — Experimental schematic in vivo and in vitro. (A) Experimental design diagram of the Sham group and the 12 h, 24 h, and 3 days groups after SAH. (B) Experimental design diagram to explore signaling pathways in vivo. (C) Experimental design diagram to explore signaling pathways in vitro. SAH, subarachnoid hemorrhage; OxyHb, oxyhemoglobin; AS, astrocyte; WB, Western blot; IF, immunofluorescence staining; H2O2, hydrogen peroxide; NO, nitric oxide; LSCI, laser speckle contrast imaging; GSK, GSK2795039; LV, lentivirus; AAV, adeno-associated virus. [file Image_1.TIF]

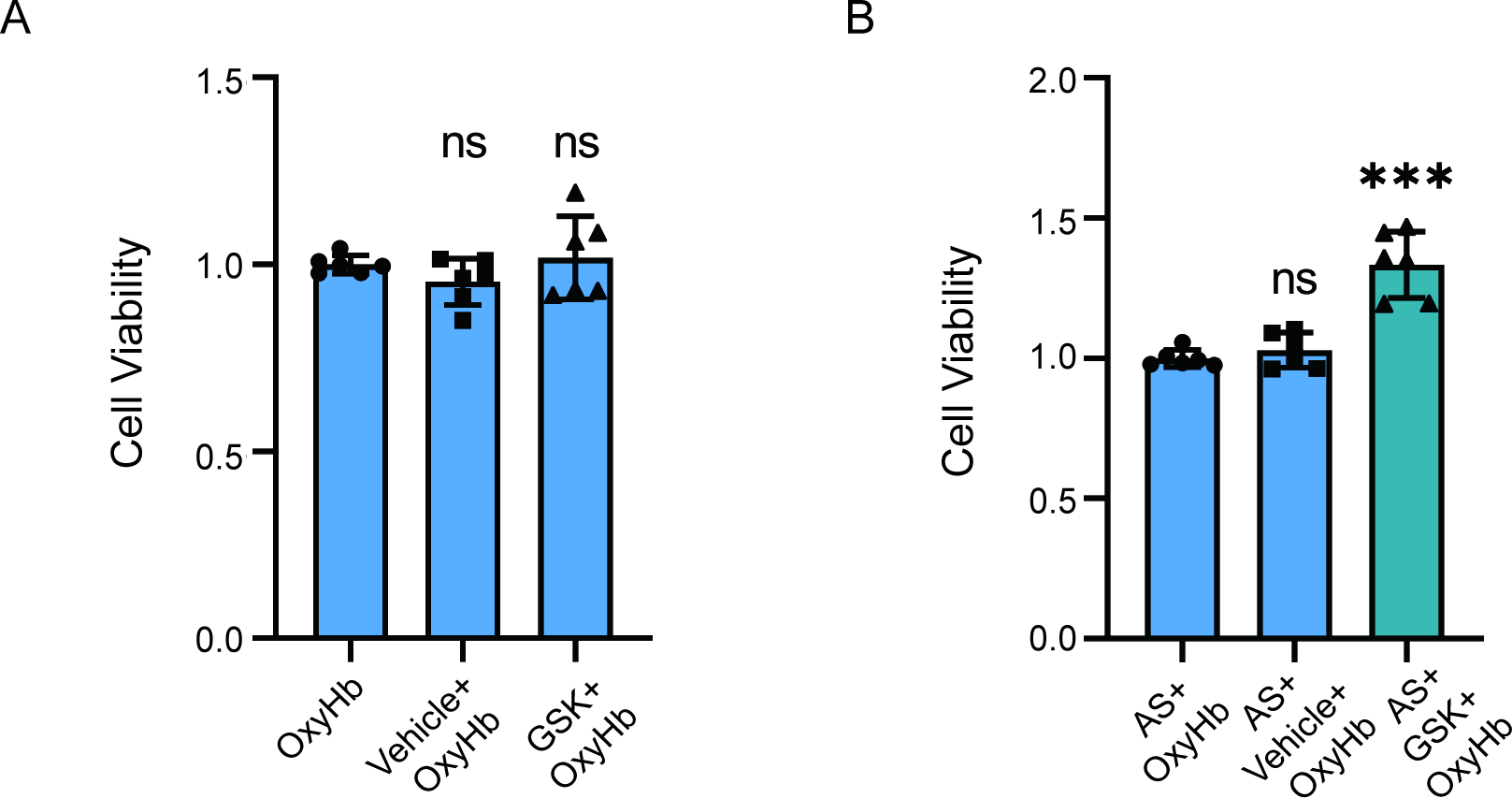

Supplement: Supplementary Figure 2 — The viability of different groups of astrocytes and endothelial cells. (A) Astrocyte viability among different experimental groups. Data are shown as mean ± SD (n = 6 each group, ns: no statistically significant difference). (B) Endothelial cell viability among different experimental groups. Data are shown as mean ± SD (n = 6 each group, ***p < 0.001, ns: no statistically significant difference). [file Image_2.TIF]

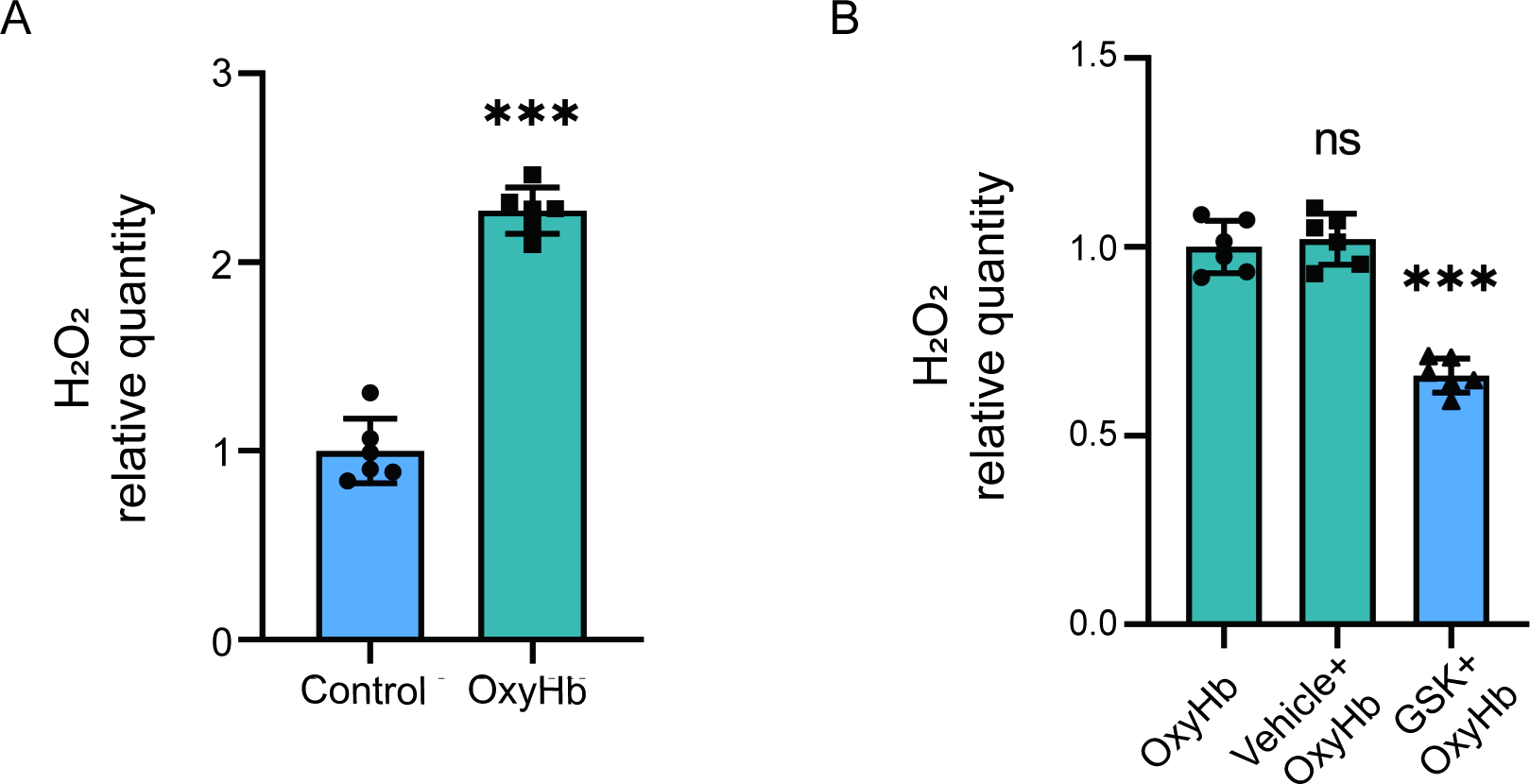

Supplement: Supplementary Figure 3 — The content of H2O2 released by astrocytes in the medium. (A) H2O2 released by astrocytes among different experimental groups. Data are shown as mean ± SD (n = 6 each group, ***p < 0.001). (B) H2O2 released by astrocytes among different experimental groups. Data are shown as mean ± SD (n = 6 each group, ***p < 0.001, ns: no statistically significant difference). [file Image_3.TIF]
